# Scalable quantum processors empowered by the Fermi scattering of Rydberg electrons

**DOI:** 10.1038/s42005-023-01174-4

**Published:** 2023-03-31

**Authors:** Mohammadsadegh Khazali, Wolfgang Lechner

**Affiliations:** 1 Institute for Quantum Optics and Quantum Information of the Austrian Academy of Sciences, A-6020 Innsbruck, Austria; 2 School of Physics, Institute for Research in Fundamental Sciences (IPM), Tehran, 19395-5531 Iran; 3 Department of Physics, University of Tehran, 14395-547 Tehran, Iran; 4 Institute for Theoretical Physics, University of Innsbruck, A-6020 Innsbruck, Austria; 5 Parity Quantum Computing GmbH, A-6020 Innsbruck, Austria

**Keywords:** Quantum information, Quantum simulation, Quantum information

## Abstract

Quantum computing promises exponential speed-up compared to its classical counterpart. While the neutral atom processors are the pioneering platform in terms of scalability, the dipolar Rydberg gates impose the main bottlenecks on the scaling of these devices. This article presents an alternative scheme for neutral atom quantum processing, based on the Fermi scattering of a Rydberg electron from ground-state atoms in spin-dependent lattice geometries. Instead of relying on Rydberg pair-potentials, the interaction is controlled by engineering the electron cloud of a sole Rydberg atom. The present scheme addresses the scaling obstacles in Rydberg processors by exponentially suppressing the population of short-lived states and by operating in ultra-dense atomic lattices. The restoring forces in molecule type Rydberg-Fermi potential preserve the trapping over a long interaction period. Furthermore, the proposed scheme mitigates different competing infidelity criteria, eliminates unwanted cross-talks, and significantly suppresses the operation depth in running complicated quantum algorithms.

## Abstract (web summary)

Current Rydberg quantum processors suffer from limitations in fidelity and scalability. Here, the authors address these limitations by proposing highly controllable multi-qubit operations that utilize the Fermi scattering of Rydberg electrons trapped in an optical lattice.

## Introduction

In the transition from the noisy intermediate scale quantum (NISQ) devices, the neutral atom quantum processors are the pioneering platform in terms of scalability with capacities up to 324 qubits^1–4^. The current limit on the size of optical lattices is imposed by the available laser powers, calling for denser lattice configurations. While accommodating orders of magnitude more atoms in the same lattice area is possible, the minimum interatomic distance of a few micrometers are used in Rydberg dipolar processors^5–7^. This limit is imposed to avoid level mixing and to preserve the trapping under strong interaction. Considering the new advances in sub-wavelength imaging, trapping, and laser addressing of atoms^8–15^, new types of interaction that operates at small interatomic distances are quite demanding.

Another bottleneck in the scalability of neutral atom processors is the short lifetime of the Rydberg states. The corresponding error scales with the number of qubits and the depth of algorithm to the limit that fails the operation of complicated tasks. The correction capabilities come with encoding the logical qubits in multiple physical qubits and are protected by error-correction codes^16,17^. This would significantly downgrade the qubit numbers that are available for logical operation. Furthermore, quantum operations on the logic level are performed by an overload of operations at the level of physical qubits and require costly techniques^17–23^. This would dramatically increase the required computational power. The alternative solution is to use system-specific properties to suppress the Rydberg population over running complicated algorithms.

The other approach to harness maximum computational power from the current NISQ processors is by circumventing the circuit model. In this approach the system-specific characteristics are deployed to directly run complicated algorithms instead of performing concatenation of one- and two-qubit gates^24,25^ with significant overhead^26,27^. In this regard, long-range many-body Rydberg interaction is vastly used for direct implementation of multi-qubit gates^28–35^, as well as other parallel-operations in different quantum processing schemes^36–43^. In an architecture of one vs many qubits, the presence/absence of inter/intra component interaction paves the way for parallel computation. Some examples include the fast implementation of C_*k*_-NOT^28–31^ and C-NOT^*k *^^29^ gates with minimal steps as well as direct operations in logical basis^44^. The parallel operation of multi-qubit gates in the Rydberg-dipolar scheme is carried out by exciting all *k* control atoms in $\left\vert {0}_{c}\right\rangle$ state to the Rydberg level followed by target Rydberg rotation^29–31^. This results in competing requirements i.e. to preserve the lattice trapping against strong dipolar interaction, to overcome/preserve the blockade between inter/intra components, and to avoid exciting the neighboring Rydberg levels^28–30,45–47^. More importantly, the unwanted intra-componant interaction disrupts the operation for specific qubit configurations, see Discussions.

There are two prominent multi-qubit operations that a full-fledged quantum processor must be able to perform with minimal steps. The Toffoli closes a set of universal gates next to the available Hadamard rotation^48^. It also plays a pivotal role in quantum error correction^49,50^, fault-tolerant quantum computation (QC)^51,52^, and Shore’s algorithm^53^. The other prominent gate, the stabilizer, is vastly used for running different quantum algorithms namely Kitaev’s toric code^54^, color code^55^, and quantum optimization problems^56,57^. For example, the parity architecture^56,57^ translates a problem with all to all connectivity to a simple nearest-neighbor problem-independent interaction. Hence, the quantum approximate optimization algorithm (QAOA)^58,59^ implementation would be simplified to programming single-qubit operations, as well as applying problem-independent four-body stabilizer-phase gate.

In search of an alternative interaction to empower quantum computation, a distinguished choice is the Fermi scattering of the Rydberg electron from other ground-state atoms embedded in the wave function of this atomic giant. This phenomenon has been widely studied in the context of Rydberg molecules in the Bose-Einstein condensate (BEC)^60–64^. The main challenge in deploying this interaction for quantum computation is to make the interaction qubit-dependent. A previous study observed the spin-flip via Rydberg-Fermi interaction at very short inter-atomic distances of ~30 nm in BEC^65^. However, above 50 nm the scattering interaction would not be spin-dependent, depriving the implementation of the scheme in a realistic atomic lattice.

This paper deploys dual spin/spatial encoding to harness the Fermi scattering of a Rydberg electron from neighboring lattice sites as a source of qubit-dependent interaction. The spin-dependent geometrical shift of atoms^66–75^ accommodates them inside or outside of the Rydberg electron cloud, generating the desired contrast of scattering interaction between different qubit configurations and thus, performing the desired quantum operation. Regarding the scalability, the scheme operates at significantly densified atomic lattices with exponentially suppressed Rydberg population compared to the dipolar counterparts. The system-specific nature of the scheme allows for performing multi-qubit operations on the nearest neighbor qubits by exciting a sole atom in a single step. Thus, it operates at a different regime of energy hierarchy without the mentioned rivalry in Rydberg-dipolar systems. Furthermore, the absence of intra-component interaction eliminates the unwanted phase errors in multi-qubit gates, see Discussion. These characteristics promises running complicated algorithms with current experimental limitations.

## Results

### Rydberg-Fermi interaction in a qubit-dependent lattice

**Fig. 1:**
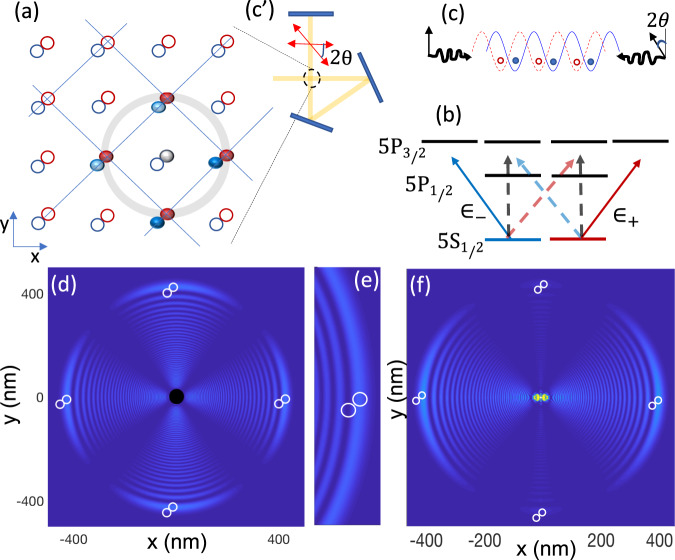
Rydberg-Fermi interaction in Spin-dependent lattice. **a** In a 2D structure with a single atom per site, applying a qubit-dependent lattice-shift makes each atom in a spatial superposition of being in red and blue sites where the components are controlled by the internal electronic qubit-states $\left\vert 0\right\rangle$ and $\left\vert 1\right\rangle$. Hence the Rydberg electron of the central atom would exclusively scatter from the plaquette atoms in a specific spin-lattice, providing qubit-dependent interaction. **b** In ^87^Rb, tuning the trapping laser between 5*P*_3/2_ and 5*P*_1/2_, the polarizability of qubit states $\left\vert 0\right\rangle$ and $\left\vert 1\right\rangle$ are given by distinguished left *ε*_−_ and right *ε*_+_ circularly polarized lights respectively. **c**, **c'** Counter propagating linearly polarized lights with relative polarization of 2*θ*, form two distinguished optical-lattices of *ε*_−_ and *ε*_+_ displaced by *D*_{*x*, *y*}_ = 2*θ*/*k* in each dimension, trapping different qubit states. **d** Exciting the Rydberg superposition state of Eq. (4) with n=64 with the quantization axis being perpendicular to the lattice plane provides a symmetric interaction over the four neighboring plaquette atoms. **e** The zoomed vision shows that the $\left\vert 0\right\rangle$ and $\left\vert 1\right\rangle$ qubit states of the plaquette atoms are localized on the node and anti-node of the Rydberg wave-function, providing a qubit-dependent contrast of Fermi scattering. **f** Two-color excitation of $(\vert 65{P}_{3/2},1/2\rangle +\left\vert 65{P}_{1/2},1/2\right\rangle )/\sqrt{2}$ with in-plane quantization axis provides couplings with two opposite plaquette atoms in the lattice.

**Fig. 2:**
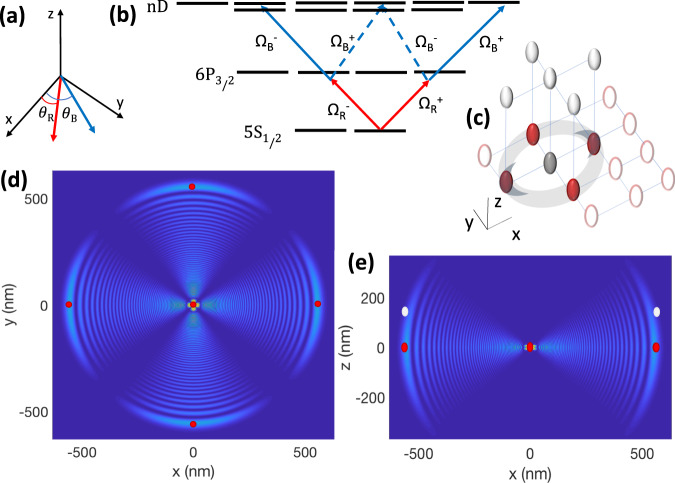
Rydberg superposition states. **a** The desired Rydberg superposition could be controlled by the polarization angles *θ*_*R*,*B*_ of the two linearly polarized fields with Rabi-frequencies Ω_*R*,*B*_ propagating along the *z* direction. **b** Decomposing each of the linearly polarized lights Ω in terms of right Ω^+^ and left Ω^−^ circularly polarized elements, the transitions shown by dashed lines would form destructive interference when *θ*_*R*_ − *θ*_*B*_ = *π*/2, leading to the superposition of $({{{{{{{{\rm{e}}}}}}}}}^{{{{{{{{\rm{i}}}}}}}}({\theta }_{R}+{\theta }_{B})}\left\vert {m}_{j}=5/2\right\rangle +{{{{{{{{\rm{e}}}}}}}}}^{-{{{{{{{\rm{i}}}}}}}}({\theta }_{R}+{\theta }_{B})}\left\vert {m}_{j}=-3/2\right\rangle )/\sqrt{2}$ states of 74D Rydberg level. **c** Applying the qubit-dependent shift perpendicular to the lattice plane allows tunning the in-plain inter-atomic distance. The **d** *xy* and **e** *xz* cross-sections of Rydberg-Fermi interaction. The excited Rydberg superposition state would be further confined around the position of plaquette atoms and hence enhances the interaction. Red and White ovals present the qubit-dependent position of $\left\vert 0\right\rangle$ and $\left\vert 1\right\rangle$ states.

In a two-dimensional lattice shown in Fig. 1a, atoms in spin-states $\left\vert 0\right\rangle$, $\left\vert 1\right\rangle$ are trapped in shifted lattices distinguished by red and blue. The gate operations are carried out by exciting the central atom in a plaquette to the Rydberg level. Depending on whether the central atom is excited from $\left\vert 0\right\rangle$ or $\left\vert 1\right\rangle$ state, the plaquette atoms in $\left\vert 0\right\rangle$ or $\left\vert 1\right\rangle$ spin-lattices would be localized on the nodes and antinodes of the Rydberg electron’s last lobe, see Fig. 1d–f. This provides contrast on the Fermi scattering of the electron from distinguished qubit states of plaquette atoms. The qubit-dependent interaction could also be realized by the spin-dependent shift perpendicular to the lattice plane as depicted in Fig. 2c.

The *spin-dependent lattice* is formed by counter-propagating linearly polarized lights, see Fig. 1b, c. Introducing a relative shift between the fields’ polarizations of 2*θ*, the total electric field can be written in terms of the sum of right and left circularly polarized lights $E={E}_{0}\exp (-i\nu t)({\varepsilon }_{+}\sin (kz+\theta )+{\varepsilon }_{-}\sin (kz-\theta ))$. To make a spin-dependent lattice-shift, the spin polarizabilities should be linked to different circular polarization components of lights^66^. To cancel the polarizabilities with unwanted light elements shown by dashed lines in Fig. 1b, the trapping laser must be tuned between *P*_3/2_ and *P*_1/2_ states so that the ac-Stark shifts of these two levels cancel each other. As a result the *m*_*j*_ = ± 1/2 levels of the ground state would be trapped by ${V}_{\pm }=\alpha | {E}_{0}{| }^{2}\sin (kz\pm \theta )$. The hyperfine qubit states $\left\vert 0\right\rangle =\left\vert F=1,{m}_{F}=1\right\rangle$ and $\left\vert 1\right\rangle =\left\vert F=2,{m}_{F}=2\right\rangle$ experience the trapping potentials *V*_0_ = (*V*_+_ + 3*V*_−_)/4 and ${V}_{\left\vert 1\right\rangle }={V}_{+}$, see Supplementary Note 1 for alternative encodings. A spin-dependent lattice provides dual spin/spatial encoding of the qubit. A Raman transition coherently transfers atoms from one internal state to the other, thereby causing hopping between the two Wannier-functions^76–78^. The spin rotation Rabi frequency in qubit-dependent lattice would be modified by the Frank-Condon factor, see Methods.

#### Ryd-Fermi Interaction

The interaction between the Rydberg electron and the ground state atom is a Fermi-type pseudo potential^79–81^,

1 $${V}_{{{{{{{{\rm{RF}}}}}}}}}=\left(2\pi \frac{\tan ({\delta }^{s})}{k(R)}-6\pi \frac{\tan ({\delta }^{p})}{{k}^{3}(R)}{\overleftarrow{\nabla }}_{{{{{{{{\bf{r}}}}}}}}}.{\overrightarrow{\nabla }}_{{{{{{{{\bf{r}}}}}}}}}\right)\delta ({{{{{{{\bf{r}}}}}}}}-{{{{{{{\bf{R}}}}}}}})$$

with **r** and **R** being the positions of the Rydberg electron and the ground state atom with respect to the ionic core, and *δ*^{*s*, *p*}^ are the triplet s- and p-wave scattering phase shift of the Rydberg electron from the neighboring ground state atom^82^. The electron wave-vector *k* is defined by the kinetic energy of the Rydberg electron at energy *E* = − 1/2*n*^2^ when it collides with a ground-state atom at position *R* from the ionic core, i.e. *k*^2^(*R*)/2 = *E* + 1/*R*. The level-shift caused by the Rydberg electron scattering from the *l*^th^ plaquette atom in the qubit state $i=\left\vert 0,1\right\rangle$ would be characterized by two parameters

2 $${\bar{V}}_{{{{{{{{\rm{RF}}}}}}}}\left\vert {i}_{l}\right\rangle }= 	 \int| w({{{{{{{\bf{R}}}}}}}}-{{{{{{{\bf{{l}}}}}}}_{i}}}){| }^{2}{V}_{RF}({{{{{{{\bf{R}}}}}}}}){{{{{{{\rm{d}}}}}}}}{{{{{{{\bf{R}}}}}}}}\\ {{{{{{{{\rm{MD}}}}}}}}}_{{V}_{{{{{{{{\rm{RF}}}}}}}}\left\vert {i}_{l}\right\rangle }}= 	 \int| w({{{{{{{\bf{R}}}}}}}}-{{{{{{{\bf{{l}}}}}}}_{i}}}){| }^{2}| {V}_{RF}({{{{{{{\bf{R}}}}}}}})-{\bar{V}}_{{{{{{{{\rm{RF}}}}}}}}\left\vert {1}_{l}\right\rangle }| {{{{{{{\rm{d}}}}}}}}{{{{{{{\bf{R}}}}}}}}$$

that are the average and the mean deviation of the scattering energy over the *l*^*th*^ plaquette atom’s Wannier-state *w* centered at *l*_*i*_. Considering the wave-function’s symmetry of the centered Rydberg atom, all surrounding plaquette atoms would experience the same qubit-dependent interactions, see Fig. 1a, d and Fig. 2c, d. The Rydberg electron’s wave-packet dynamics are in the ps range^83,84^. Therefore, over the MHz scale of operation, the interaction of the Rydberg electron with all the plaquette atoms in $\left\vert 1\right\rangle$ qubit state would be alike and add up^64^.

Two-color excitation of the superposition state $(\vert 65{P}_{3/2},1/2\rangle +\vert 65{P}_{1/2},1/2\rangle )/\sqrt{2}$ with in-plane quantization axis provides sites’ specific couplings along a line, see Fig. 1f. The two-color light could be obtained in a setup of beamsplitters and acusto-optical modulators. The generated superposition mainly contains the *Y*_1,0_ spherical harmonic term, which significantly concentrates the electron wave-function along the quantization axis and hence enhances the interaction strength. The atoms prepared in the ground motional state^85–88^ are considered delocalized over the Gaussian wave-function. Hence they would experience an effective Rydberg-Fermi interaction that is averaged over their spatial profile. The scattering energy of Rydberg electron over the qubit-dependent Wannier-state of the l^*th*^ plaquette atom with FWHM = 20 nm would be quantified by Eq. (2) as

3 $${\bar{V}}_{{{{{{{{\rm{RF}}}}}}}}\left\vert {1}_{l}\right\rangle }= 	 \, 2\,{{{{{{{\rm{MHz}}}}}}}}, \quad{{{{{{{{\rm{MD}}}}}}}}}_{{V}_{{{{{{{{\rm{RF}}}}}}}}\left\vert {1}_{l}\right\rangle }}=0.1\,{{{{{{{\rm{MHz}}}}}}}}\\ {\bar{V}}_{{{{{{{{\rm{RF}}}}}}}}\left\vert {0}_{l}\right\rangle }= 	 \, 0.3\,{{{{{{{\rm{MHz}}}}}}}}, \quad{{{{{{{{\rm{MD}}}}}}}}}_{{V}_{{{{{{{{\rm{RF}}}}}}}}\left\vert {0}_{l}\right\rangle }}=0.2\,{{{{{{{\rm{MHz}}}}}}}}$$

where the qubit-dependent lattice-shift of *D* = 36.8 nm is considered.

To apply a uniform interaction on all the plaquette atoms, the polarization axis must be perpendicular to the lattice plane, see Fig. 1d and 2d. The interaction enhancement can be obtained by exciting superposition of Rydberg levels. The spatial constructive (destructive) interference of Rydberg wave-functions over the position of plaquette qubits (elsewhere) could further confine the electron and hence enhances the interaction. The desired Rydberg superposition could be controlled by the polarization angles *θ*_{*R*, *B*}_ of the two linearly polarized lights Ω_{*R*, *B*}_ used for Rydberg excitations, see Fig. 2. These fields are propagating perpendicular to the lattice plane along the *z* direction. The linear polarized light could be expressed in terms of circular polarizations ${{{\Omega }}}_{j}=(\exp ({{{{{{{\rm{i}}}}}}}}{\theta }_{j}){{{\Omega }}}_{j}^{+}+\exp (-{{{{{{{\rm{i}}}}}}}}{\theta }_{j}){{{\Omega }}}_{j}^{-})/\sqrt{2}$. Adjusting *θ*_*R*_ − *θ*_*B*_ = *π*/2 the transition to $\vert n{D}_{j},{m}_{j}=1/2\rangle$ would be canceled by destructive interference. Hence the excited state would be

4 $${e}^{{{{{{{{\rm{i}}}}}}}}({\theta }_{R}+{\theta }_{B})}\frac{\left\vert n{D}_{\frac{5}{2}},\frac{5}{2}\right\rangle }{\sqrt{2}}+{{{{{{{{\rm{e}}}}}}}}}^{-{{{{{{{\rm{i}}}}}}}}({\theta }_{R}+{\theta }_{B})}\frac{\left\vert n{D}_{\frac{5}{2}},\frac{-3}{2}\right\rangle +\left\vert n{D}_{\frac{3}{2}},\frac{-3}{2}\right\rangle }{2}.$$

The simultaneous excitation of both $\vert n{D}_{3/2}\rangle$ and $\vert n{D}_{5/2}\rangle$ could be obtained by beam splitting and frequency adjustment of the blue laser by acusto-optical modulators. The polarization angles in Eq. (4) would act as a controlling parameter to rotate the interaction maxima e.g. *θ*_*R*_ + *θ*_*B*_ = *π*, 0 are corresponding to Fig. 2d and the same pattern rotated by *π*/4 around the $\hat{z}$ axis. For the sake of presentation, Fig. 1 and 2 only plot the s-wave scattering part of *V*_RF_, which is the dominant term at the desired last lobe in ^87^Rb atoms. The exciting laser’s polarization and propagation direction could act as a controlling knob to program different interaction connectivities among neighboring lattice sites.

In the setup of Fig. 1d with the lattice constant of 400nm, exciting the central atom to the superposition state of Eq. (4) with *n* = 64, the scattering energy of the Rydberg electron over the qubit-dependent Wannier-state of the l^*th*^ plaquette atom with FWHM_*x*,*y*_ = 20 nm and FWHM_*z*_ = 35 nm would be quantified by Eq. (2) as

5 $${\bar{V}}_{{{{{{{{\rm{RF}}}}}}}}\left\vert {1}_{l}\right\rangle }= 	 \, 2.5\,{{{{{{{\rm{MHz}}}}}}}}, \quad{{{{{{{{\rm{MD}}}}}}}}}_{{V}_{{{{{{{{\rm{RF}}}}}}}}\left\vert {1}_{l}\right\rangle }}=0.25\,{{{{{{{\rm{MHz}}}}}}}}\\ {\bar{V}}_{{{{{{{{\rm{RF}}}}}}}}\left\vert {0}_{l}\right\rangle }= 	 \, 0.35\,{{{{{{{\rm{MHz}}}}}}}}, \quad{{{{{{{{\rm{MD}}}}}}}}}_{{V}_{{{{{{{{\rm{RF}}}}}}}}\left\vert {0}_{l}\right\rangle }}=0.2\,{{{{{{{\rm{MHz}}}}}}}},$$

where the magic qubit-dependent lattice-shift of *D* = 35.6 nm results in uniform ${\bar{V}}_{{{{{{{{\rm{RF}}}}}}}}\left\vert {0}_{l}\right\rangle }$ inside and outside the last lob. To further narrow the interaction-induced line broadening ${{{{{{{{\rm{MD}}}}}}}}}_{{V}_{{{{{{{{\rm{RF}}}}}}}}\left\vert {i}_{l}\right\rangle }}$, ultra-tight confinement could be obtained in quantum-twist optical-lattices^14^.

**Fig. 3:**
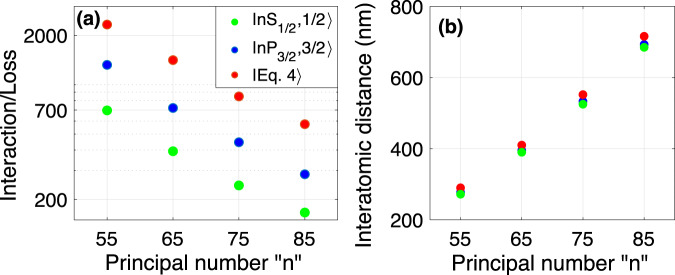
Scaling of interaction-to-loss ratio. **a** The scattering interaction of Rydberg electron from the four plaquette atoms over the decay rate of the central Rydberg atom is plotted as a function of the principal number for Rydberg states $\vert n{S}_{1/2},1/2\rangle$, $\vert n{P}_{3/2},3/2\rangle$, and for the state presented in Eq. (4). **b** While operating at smaller *n* enhances the coherence, it requires smaller inter-atomic distances, raising concerns about single-site addressing, see Methods.

The scale of interaction to loss ratio improves by going to smaller Rydberg principal numbers. The volume of the Rydberg electron’s wave-function scales by *n*^6 ^^81^. Hence the electron density and the interaction scales by *V*_RF_ ∝ *n*^−6^. Since the lifetime of the Rydberg state scales by *n*^3 ^^89^, the interaction to loss ratio would scale by *n*^−3^, see Fig. 3a. While the lattice configuration of Fig. 2c allows tuning the desired inter-atomic distance, going to lower principal numbers would raise the errors related to single site addressing, see Fig. 3b and Methods. However, the new advances in sub-wavelength trapping, spin rotating, and imaging^8–14^ alleviate the current limits and provide a wide range of opportunities for the Rydberg-Fermi QC. Also, dual-species lattices^56,90–92^ could be used to suppress the laser-cross talk issues in compact lattices.

Applying the lattice shift perpendicular to the lattice plane provides freedom in choosing the inter-atomic distance, see Fig. 2c–e. In the *λ* = 1064 nm optical lattice^13^, exciting the central atom to the superposition state of Eq. (4) with *n* = 74, the scattering energy of the Rydberg electron over the qubit-dependent Wannier-state of the l^*th*^ plaquette atom with FWHM_*x*,*y*_=25 nm and FWHM_*z*_ = 35 nm would be quantified by Eq. (2) as

6 $${\bar{V}}_{{{{{{{{\rm{RF}}}}}}}}\left\vert {1}_{l}\right\rangle } = 	 \, 1.1\,{{{{{{{\rm{MHz}}}}}}}}, \quad{{{{{{{{\rm{MD}}}}}}}}}_{{V}_{{{{{{{{\rm{RF}}}}}}}}\left\vert {1}_{l}\right\rangle }}=0.07\,{{{{{{{\rm{MHz}}}}}}}}\\ {\bar{V}}_{{{{{{{{\rm{RF}}}}}}}}\left\vert {0}_{l}\right\rangle } = 	 \, 0.1\,{{{{{{{\rm{MHz}}}}}}}}, \quad{{{{{{{{\rm{MD}}}}}}}}}_{{V}_{{{{{{{{\rm{RF}}}}}}}}\left\vert {0}_{l}\right\rangle }}=0.05\,{{{{{{{\rm{MHz}}}}}}}},$$

where the qubit-dependent lattice-shift of *D*_*z*_ = 180 nm is considered.

### Implementation of multi-qubit gates

#### Parallelized gate

The Ryd-Fermi interaction in qubit-dependent atomic lattice could be used for the implementation of the (C-NOT^*k*^) parallelized gate

7 $${U}_{g}={\left\vert 0\right\rangle }_{c}\left\langle 0\right\vert \otimes {\mathbb{I}}+{\left\vert 1\right\rangle }_{c}\left\langle 1\right\vert \otimes \mathop{\prod }\limits_{i=1}^{4}{\sigma }_{x}^{i}$$

which is an essential element in realizing the stabilizer-phase gates^42^, see Methods for a detailed discussion.

In this proposal, the target atoms are localized on the square plaquette around the central control atom. Exciting the $\vert {1}_{c}\rangle$ state of the control atom to the Rydberg level, its electron creates potential energy shits via Fermi scattering. In the spin-dependent lattice, the contrast of scattering energy depends on the presence or absence of the Rydberg electron at the position of different qubit states. Over the operation time *τ*, the effective Hamiltonian

8 $$H={a}_{{c}_{1}}^{{{{\dagger}}} }{a}_{{c}_{1}}\mathop{\sum}\limits_{l\in p}({\bar{V}}_{{{{{{{{\rm{RF}}}}}}}}\left\vert {1}_{l}\right\rangle }{a}_{{l}_{1}}^{{{{\dagger}}} }{a}_{{l}_{1}}+{\bar{V}}_{{{{{{{{\rm{RF}}}}}}}}\left\vert {0}_{l}\right\rangle }{a}_{{l}_{0}}^{{{{\dagger}}} }{a}_{{l}_{0}})$$

accumulates a contrast of *π* phase on each target atom in $\left\vert 1\right\rangle$ qubit state conditioned on the control atom being in state $\vert {1}_{c}\rangle$. Here ${a}_{{l}_{i}}^{({{{\dagger}}} )}$ annihilates (creates) the Wannier-state of the *l*th target qubit in the plaquette, centered at *l*_*i*_ with *i* ∈ {0, 1} defining the qubit-dependent trap. Compensating the background phase and applying Hadamard to the individual target atoms before and after the Rydberg excitation results in the desired operation of Eq. (7).

*Gate fidelity:* The main sources of errors in quantifying the C-NOT^4^ gate’s operation are spontaneous emission and population rotation errors. The errors are averaged over the 2^5^ qubit configurations. The average spontaneous emission error from the Rydberg level is given by ${E}_{sp,r}=\frac{1}{2}\frac{\pi }{{U}_{{{{{{{{\rm{RF}}}}}}}}}}{{{\Gamma }}}_{{{{{{{{\rm{r}}}}}}}}}$, where *U*_RF_ is the qubit-dependent contrast of ${\bar{V}}_{{{{{{{{\rm{RF}}}}}}}}}$ and Γ_r_ is the decay rate of the Rydberg state^89^. In a two-photon excitation scheme of Fig. 2a, b, partial population of the intermediate $\vert 6{P}_{3/2}\rangle$ level results in an extra source of loss. Considering the effective two-photon excitation ${{{\Omega }}}_{r}=\frac{{{{\Omega }}}_{420}{{{\Omega }}}_{1013}}{2{\delta }_{p}}$, using high power lasers^93^ facilitates large Rabi frequencies Ω_1013_/2*π* = 250 MHz, Ω_420_/2*π* = 250 MHz and *δ*_*p*_/2*π* = 5 GHz. The corresponding average error would be ${E}_{se,p}=\frac{\pi }{4{\tau }_{p}{\delta }_{p}}(q+1/q)=4\times 1{0}^{-4}$ ^94^ with intermediate level lifetime of *τ*_*p*_ = 113 ns and *q* = Ω_420_/Ω_1013_. The control atom’s rotation error is due to the unwanted excitation of neighboring accessible Rydberg levels separated in energy by *δ*_*r*_/2*π* = 17 GHz, 21 GHz, and 6 GHz in Fig. 1f, d and 2 respectively. The corresponding error averaged over qubit configurations would be $1/2\frac{{{{\Omega }}}_{r}^{2}}{4{\delta }_{r}^{2}}$. Finally, non-deterministic excitation of the Rydberg atom due to qubit-dependent level-shift caused by Rydberg-Fermi interaction should be overcome by the strong exciting laser Ω_*r*_ tuned to the middle of the spectrum leading to the average error of $$1/{2}^{5}{\sum }_{j=0}^{4}\big(\begin{array}{c}4\\ j\end{array}\big)\frac{{(j-2)}^{2}{U}_{RF}^{2}}{{{{\Omega }}}_{r}^{2}}$$. Overall, using the schemes described in Fig. 1f, 1d, 2d with interactions quantified in Eq. 3, 5, 6 and with the respective Rabi-frequencies Ω_*r*_ = 200, 100, 40 MHz results in high fidelity fan-out gate with the average fidelity of F = 99.8%, 99.7%, 99.6% at the cryogenic environment of 77 K and *F* = 99.5%, 99.3%, 99.2% without cryogenic environment at 300 K. The bottleneck in operation fidelity comes from the small lifetime of the Rydberg level. Using Rydberg circular state enhances the interaction-to-loss ratio by four orders of magnitude, see Methods.

#### Toffoli gate

The *Toffoli* gate C_*k*_-NOT with *k* = 4, 6 could be realized in square and triangular lattices, by placing the control atoms over the plaquette and exciting the central target atom in $\left\vert {1}_{t}\right\rangle$ state to the Rydberg level. The Fermi scattering of the Rydberg electron from control atoms forms an interaction-based level-shift on the target atom that depends on the spatial qubit configuration of the entire system. Unlike the C-NOT^*k*^ gate, Toffoli does not operate with a strong laser for deterministic Rydberg excitation. Here a weak transition ${{{\Omega }}}_{r}=\frac{{{{\Omega }}}_{420}{{{\Omega }}}_{1013}}{2{\delta }_{p}}\ll {V}_{{{{{{{{\rm{RF}}}}}}}}}$ would selectively excite the Rydberg atom conditioned on the control atoms to be in ${\left\vert 0\right\rangle }_{c}^{\otimes k}$ state. The presence of any $\left\vert {1}_{c}\right\rangle$ state localized that control atom inside the Rydberg wave-function of the target atom, shifting the laser out of resonance and blocking the transition. The operation Hamiltonian would be

9 $${H}_{{{{{{{{\rm{tof}}}}}}}}} =\, 	 ({{{\Omega }}}_{1}{\sigma }_{1p}+{{{\Omega }}}_{2}{\sigma }_{rp}+h.c.)+{\delta }_{p}{\sigma }_{pp}+{{\Delta }}{\sigma }_{rr}\\ 	 +{\sigma }_{rr}\mathop{\sum}\limits_{l\in p}({\bar{V}}_{{{{{{{{\rm{RF}}}}}}}}\left\vert {1}_{l}\right\rangle }{a}_{l1}^{{{{\dagger}}} }{a}_{l1}+{\bar{V}}_{{{{{{{{\rm{RF}}}}}}}}\left\vert {0}_{l}\right\rangle }{a}_{l0}^{{{{\dagger}}} }{a}_{l0})$$

where ${\sigma }_{ij}=\left\vert i\right\rangle \langle j\vert$ is the transition/projection operator acting on the target atom, Ω and *δ*_*p*_, Δ are the Rabi frequency, and laser detuning from the intermediate and Rydberg levels in a two-photon excitation. The last term would sum over the control qubits around the central target atoms and apply qubit-dependent Rydberg-Fermi interaction. Adjusting the laser to ${{\Delta }}=-k{\bar{V}}_{{{{{{{{\rm{RF}}}}}}}}\left\vert {0}_{l}\right\rangle }$, the 2*π* rotation of the target atom would be conditioned on the presence of $\left\vert {0}_{c}^{k}{1}_{t}\right\rangle$ state, generates a *π* phase, and apply the desired C_*k*_-Z operation. Sandwiching the target atom with Hadamard gates results in the desired Toffoli operation. The Rydberg-Fermi interaction operates the C_*k*_-Z with a single 2*π* pulse addressing the Rydberg level, leaving no Rydberg population unprotected from the laser. This would eliminate the errors associated with the conventional gate schemes with *π*-gap-*π* Rydberg exciting pulses as discussed in^95–97^.

**Fig. 4:**
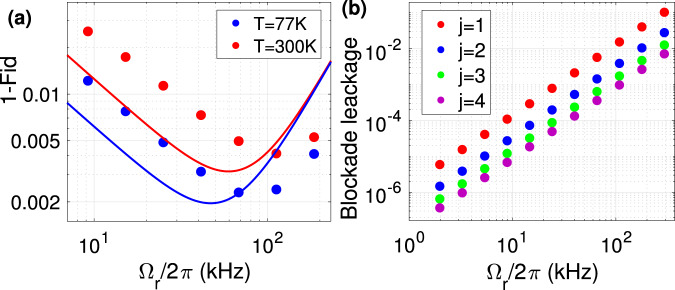
Optimal gate performance. **a** The infidelity of C_4_-NOT operation as a function of Rydberg exciting Rabi-frequency for the setup of Fig. 1d with the interaction values quantified in Eq. (5). The solid line is based on analytic estimates discussed in the text for a square laser pulse while the circles are based on the numerical simulation of Eq. (9), calculated for a Gaussian pulse ${{{\Omega }}}_{r}{e}^{-\frac{{(t-T/2)}^{2}}{2{\sigma }^{2}}}{e}^{-\frac{{(T/2)}^{2}}{2{\sigma }^{2}}}$ with *σ* = *T*/5 and a pulse duration *T* given by $\int\nolimits_{0}^{T}{{\Omega }}(t){{{{{{{\rm{d}}}}}}}}t=2\pi$. The fidelity quantified by Eq. (10), encounters spontaneous emission and rotation errors. Different environment temperatures of 77 K and 300 K are considered. **b** Blockade leakage of the target atom for different qubit configurations with *j* plaquette atoms in $\left\vert {1}_{c}\right\rangle$ state. In the numerics, the effective Rabi frequency ${{{\Omega }}}_{r}=\frac{{{{\Omega }}}_{420}{{{\Omega }}}_{1013}}{2{\delta }_{p}}$ in two-photon excitation is obtained by Ω_420_ = Ω_1013_ lasers that are detuned from intermediate level by *δ*_*p*_/2*π* = 5 GHz and the lasers are locked out of phase with the locking bandwidth of 1 kHz^93^. The results are averaged over the frequency profile of the pulse as discussed in Methods.

The main sources of errors in quantifying the C_*k*_-NOT gate’s operation are spontaneous emission of the atomic levels, and population rotation errors. The spontaneous emission from the Rydberg level only occurs in the qubit configuration $\vert {0}_{c}^{k}{1}_{t}\rangle$ where the single target atom gets excited to the Rydberg state. Hence the averaged operation error of ${E}_{sp,r}=\frac{1}{{2}^{k+1}}\frac{\pi }{{{{\Omega }}}_{{{{{{{{\rm{r}}}}}}}}}}{{{\Gamma }}}_{r}$ is expected. Off-resonant Rydberg excitation results in blockade leakage adding up to the average error of $${E}_{r1}=\frac{1}{{2}^{k+1}}{\sum }_{j=1}^{k}\big(\begin{array}{c}k\\ j\end{array}\big)\frac{{{{\Omega }}}_{r}^{2}}{4{j}^{2}{U}_{RF}^{2}}$$, where *j* is the number of control atoms in $\vert {1}_{c}\rangle$ qubit-dependent lattice. In the two-photon excitation, the blockade is sensitive to the locking bandwidth of the lasers, which could be made less than 1kHz^93^. Also in the case of fast operation, the large frequency bandwidth of exciting pulses might affect the blockade at the heart of the scheme. Circle signs in Fig. 4 quantify the operation of the gate by simulating the master equation encountering the spontaneous emission from intermediate and Rydberg levels as well as the de-phasing terms associated with lasers’ line-width. Also, the frequency profiles of the laser pulses are encountered in the lasers’ detuning and Rabi frequencies as discussed in Methods. The analytic and numeric simulations of Fig. 4 suggest high fidelity operations of 99.8% could be expected in the setup of Fig. 1d. Other avenues in enhancing the fidelity are discussed in Methods, which are based on improving the interaction-to-loss ratio by using the resonance scattering in Cs atoms or exciting higher orbital angular momentum quantum numbers.

### Other sources of error

#### Rydberg molecule loss channels

Rydberg-Fermi scattering could enhance the decoherence rate in BEC^98^. This decoherence only occurs at short inter-atomic distances well inside the Rydberg orbital when the attractive Rydberg-Fermi and atom-ion interaction, move the two interacting atoms to a very small separation of order 2 nm, where the binding energy of the molecules can ionize the Rydberg electron and form a $R{b}_{2}^{+}$ molecule^99^. Without the mass transport, step-wise decay or ionization of the Rydberg atom is ruled out by the quantization of the Rydberg state, as discussed and experimentally tested in^98^. This is because the small molecular binding energy at the last lobe of the Rydberg wave-function is orders of magnitude smaller than the closest Rydberg levels for the range of principal numbers applied here. The occurrence of ion-pair formation is also highly unlikely in this system^99^. In conclusion, confining the atoms by an optical-lattice at the last lobe of the Rydberg wave-function, prevents the described mass transport and completely closes the Rydberg molecule loss channels.

#### Entanglement of computational and motional states

The other source of error is the unwanted entanglement between the motional state and the qubit configurations. In the parallelized C-NOT^*k*^ gate, the Rydberg-Fermi potential modifies the optical trapping experienced by the plaquette atoms in $\left\vert {1}_{t}\right\rangle$ Wannier states. It is important to apply the changes adiabatically to avoid an unwanted entanglement between the computational and the motional states. Over the Rydberg excitation of the control atom, target atoms in $\left\vert {1}_{t}\right\rangle$ Wannier state would experience trap evolution ${U}_{trap}={U}_{op}+{P}_{{r}_{c}}(t){V}_{{{{{{{{\rm{RF}}}}}}}}}$ where *U*_*op*_ is the optical trap potential, and ${P}_{{r}_{c}}$ is the Rydberg population of the central atom. As long as the dynamic is adiabatic, i.e. ${\dot{\omega }}_{trap}\ll {\omega }_{trap}^{2}$^100^, the $\left\vert {1}_{t}\right\rangle$Wannier states can adapt continuously and stays close to the instantaneous ground motional state. For example, operating the C-NOT^*k*^ with the setup associated with Fig. 2/Eq. (6), a linear change of Rabi frequency from Ω_*r*_ = 30 MHz to Ω_*r*_ = 45 MHz would preserve the ground motional state. Unlike the parallelized gate in Toffoli, the presence of a plaquette atom in the Rydberg wave-function blocks the Rydberg excitation. Hence no bound state would be formed and the above adiabaticity discussion does not limit the Toffoli scheme. Stronger confinement of atoms in quantum-twist optical-lattices^14^, allows faster adiabatic operations. Effects of laser rotations on entangling the thermal states are also discussed in Supplementary Note 2.

## Discussion

The proposed scheme significantly improves the gate fidelity compared to Rydberg dipolar counterparts by mitigating different sources of imperfections and simplifying the process as discussed below.

The main bottleneck in the Rydberg quantum processors is the short lifetime of Rydberg levels. The Rydberg dipolar multi-qubit Toffoli C_*k*_-NOT gate could be realized by two approaches as explained in^30^. In the fast scheme, all control atoms in $\left\vert 0\right\rangle$ qubit state would get excited to the Rydberg level with strong simultaneous pulses. In that case, the average population of Rydberg levels in control atoms over the 2*k* + 1 qubit configurations would scale by k leading to an average Rydberg decay error of *k*Γ(*π*/2Ω_*c*_ + 3*π*/2Ω_*t*_) see Eq. 5 in Isenhower et al.^30^. In the other scheme operating with sequential excitation steps, the Rydberg population is limited to one due to the global Blockade effect but the 2*k* + 3 sequence of excitation would result in a long operation time leading to the same scaling of average Rydberg decay error of control atoms *k*Γ*π*/2Ω^30^. This is in contrast to the Rydberg-Fermi scheme where the only qubit configuration with a single Rydberg population is $\left\vert {0}_{c}^{k}{1}_{t}\right\rangle$ qubit state. In the other 2^*k*+1^ − 1 qubit configurations, the target laser would be out of resonance and no population would be excited. Hence the Rydberg decay error averaged over all qubit configurations would be $\frac{1}{{2}^{k+1}}\pi {{\Gamma }}/2{{\Omega }}$. Considering the Rydberg-Fermi operation in 2D and 3D triangular lattices with *k* = 6 and 18, the averaged population of Rydberg atoms would be suppressed by 2 × 10^3^ and 4 × 10^6^ times compared to dipolar implementations. This would also make the Rydberg-Fermi scheme ideal for the implementation of quantum search algorithms^101,102^.

Considering the current trap power shortage for the scalability of neutral-atom processors, densifying the atomic lattice remains a major priority and an immense challenge. In the Ryd-Fermi scheme, the interaction-to-loss ratio improves by going to smaller interatomic distances, see Fig. 3. At small inter-atomic distances, the dipolar scheme is limited to short-lived Rydberg states with low principal numbers to avoid strong level-mixing and line-broadening^103–105^. In the Rydberg-Fermi scheme, the absence of strong level-mixing around the outer shell of the Rydberg wave-function allows for choosing highly excited Rydberg states with longer lifetimes. Unlike the dipolar scheme, the restoring forces in the molecule type Rydberg-Fermi potential preserve the trapping at short interatomic distances over a long interaction period. These facts provide the advantage of operating at denser lattice geometries compared to dipolar counterparts.

One of the advantages of implementing multi-qubit operations with the single-step Rydberg-Fermi scheme is the absence of intra-component interaction. To quantify the effects of this unwanted phase on the performance of conventional Rydberg dipolar operations, the phase-sensitive form of fidelity is used

10 $${{{{{{{\rm{Fid}}}}}}}}={{{{{{{\rm{Tr}}}}}}}}(| M+M{M}^{{{{\dagger}}} }| )/2n,$$

where $M={U}_{id}^{{{{\dagger}}} }{U}_{gate}$, with *U*_*id*_ and *U*_*gate*_ representing the ideal and realistic gate operations. Dimension of the qubit configurations in C_*k*_-NOT or C-NOT^*k*^ is given by *n* = 2^*k*+1^.

Evaluating the fidelity of Rydberg-dipolar *Toffoli gate* C_4_-NOT proposed in^30^, with the phase-dependent definition of fidelity in Eq. (10) reveal the effects of unwanted phase. In the dipolar-scheme^30^, control and target atoms are getting excited to $\left\vert 60S\right\rangle$ and $\left\vert 60P\right\rangle$, with optimum laser couplings of Ω_*c*_/2*π* = 180 MHz, Ω_*t*_/2*π* = 0.8 MHz, and lattice separation of 4 μm. Simulating the gate operation under the Schrödinger equation, encountering spontaneous emission and population rotation errors in addition to the infidelity encountered by unwanted phases leads to the average infidelity of 5% quantified by Eq. (10). One should note that large phase-dependent infidelities occur in specific qubit configurations with large Rydberg population e.g. ${\left\vert 1111\right\rangle }_{c}{\left\vert 1(0)\right\rangle }_{t}$ experience 52% (12%) infidelity. In the Rydberg-Fermi approach, the absence of unwanted intra-component interaction eliminates the unwanted phase and control atoms’ rotation errors. Notably, obtained fidelities reported in Fig. 4, 7 are below the 1% infidelity threshold for surface error correction codes^106^.

The implementation of Rydberg-dipolar parallelized C-NOT^*k*^ gate^43^ is also sensitive to the intra-component interaction. In that scheme^43^, in qubit configurations with $\left\vert {0}_{c}\right\rangle$ state, each of the target atoms would follow the dark state with the Rydberg population of ${P}_{R}={(\frac{{{{\Omega }}}_{p}}{{{{\Omega }}}_{c}})}^{2}$. Here Ω_*p*_ is the Rabi-frequencies that connect the qubit basis to the intermediate level, and Ω_*c*_ couples the intermediate level with the Rydberg state, see Supplementary Note 3. For the abbreviation, the readers are referred to Muller et al.^43^ for the scheme explanation. Applying the phase-dependent definition of fidelity in Eq. (10), the dipolar Rydberg gate^43^ shows significant sensitivity to intra-component interaction, see Supplementary Figure S2. These unwanted phases directly affect the implementation of the stabilizer operator^42^. This comparison shows the importance of the complete elimination of unwanted intra-component interactions in the Rydberg-Fermi scheme.

Another advantage of having a single Rydberg atom is closing the collective decoherence channels^107,108^. Besides, the proposed Rydberg-Fermi C_*k*_-Z gate operates with a continuous 2*π* pulse, leaving no Rydberg population unprotected from the laser. This would eliminate the errors associated with the conventional schemes with *π*-gap-*π* Rydberg exciting pulses as discussed in refs. ^95–97^. Finally, the direct implementation of multi-qubit gates in this proposal would reduce the operation steps and the accumulative errors. For example, the C_6_-NOT gate operation with concatenated Rydberg C-NOT gates^26,27^ requires 112 pulses. Significant contrast obtains in the Rydberg-Fermi scheme operating by three pulses.

In the outlook, the proposed Rydberg-Fermi interaction paves the way for long-distance entanglement and direct operations among logical basis^44^.

## Methods

### Rydberg cloud engineering in a triangular lattice

#### Rydberg states with high orbital angular momentum in rubidium lattice

Going to high orbital angular momentum numbers, the centrifugal force pushes the electron away from the core towards the neighboring ground-state atoms. This could enhance the interaction strength. In the extreme limit, the maximum angular momentum *l* = *n* − 1 in the circular Rydberg state forms an ideal torus wave-function, see below.

**Fig. 5:**
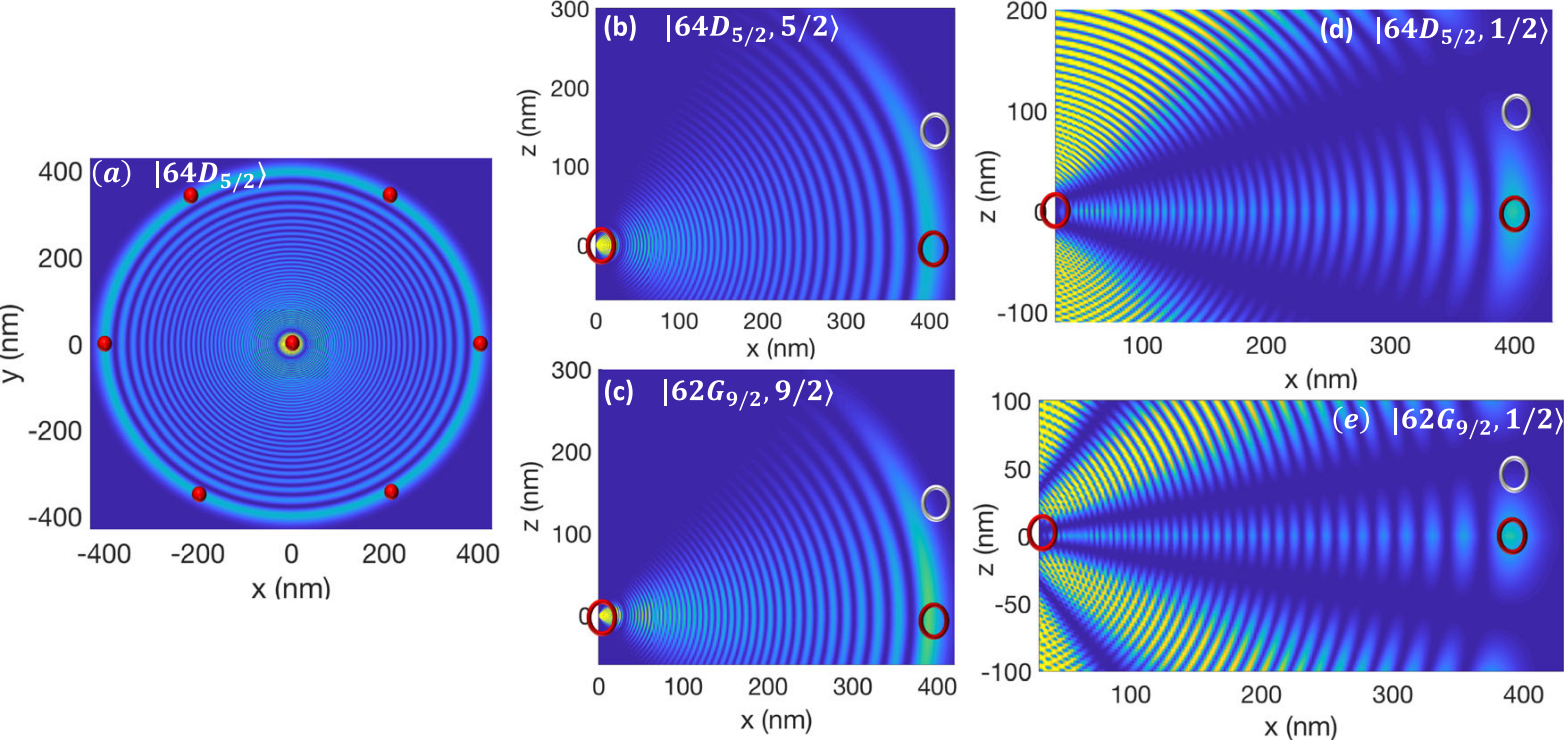
Controling the Rydberg cloud by adjusting the azimuthal and magnetic quantum numbers. The cloud geometry are depicted with respect to lattice sites presented by red/white ovals corresponding to the $\left\vert 0\right\rangle$/$\left\vert 1\right\rangle$ qubit states. **a** Shining the laser perpendicular to the lattice plane (that defines the quantization axis along *z*), the symmetry of the wave function in the lattice plane results in a uniform interaction with all plaquette qubits. **b**, **c** Exciting $\left\vert n{L}_{j},{m}_{j}=j\right\rangle$ would confine the cloud close to the lattice plane providing larger interaction. The centrifugal force at higher orbital angular momentum numbers, pushes the electron away from the core towards the neighboring ground-state atoms, enhancing the interaction. **d**, **e** Exciting $\left\vert n{L}_{j},{m}_{j}=1/2\right\rangle$ increases the number of the angular nodes, allowing a significant reduction of the qubit-dependent lattice shift with the price of reducing the interaction strength.

Exciting $\vert n{L}_{j},{m}_{j}=j\rangle$ Rydberg state would exclusively excite the *Y*_*L*,*L*_ spherical harmonic. With the quantization axis being perpendicular to the lattice, the electron wave-function would be confined close to the 2D lattice plane providing a homogenous interaction for all plaquette atoms. The electronic cloud of two Rydberg states $\vert 64{D}_{5/2},5/2\rangle$ and $\vert 62{G}_{9/2},9/2\rangle$ are plotted in Fig. 5a–c. Please note that $\left\vert nD\right\rangle$ and $\left\vert nG\right\rangle$ state could be excited via a single photon^109^ and double photon quadrupole transitions respectively. Corresponding Rydberg-Fermi interaction and gate fidelities in a triangular lattice are quantified in Table 1.

**Table 1:** Rydberg-Fermi gate operation with high orbital angular momentum Rydberg states.

#	State	*D*_*z*_	(*σ*_{*x*, *y*}_,*σ*_*z*_)	${\bar{V}}_{{{{{{{{\rm{RF}}}}}}}}\left\vert {1}_{l}\right\rangle }$	${{{{{{{{\rm{MD}}}}}}}}}_{{V}_{{{{{{{{\rm{RF}}}}}}}}\left\vert {1}_{l}\right\rangle }}$	${\bar{V}}_{{{{{{{{\rm{RF}}}}}}}}\left\vert {0}_{l}\right\rangle }$	${{{{{{{{\rm{MD}}}}}}}}}_{{V}_{{{{{{{{\rm{RF}}}}}}}}\left\vert {0}_{l}\right\rangle }}$	1-Fid
		(nm)	(nm,nm)	(MHz)	(MHz)	(MHz)	(MHz)	C_6_-NOT
1	$\left\vert 64{D}_{5/2},5/2\right\rangle$	150	(25,30)	1.2	0.13	0.17	0.09	0.002
2	$\left\vert 62{G}_{9/2},9/2\right\rangle$	150	(25,30)	1.7	0.17	0.16	0.08	-
3	$\left\vert 64{D}_{5/2},1/2\right\rangle$	100	(25,30)	0.45	0.047	0.04	0.015	0.003
4	$\left\vert 62{G}_{9/2},1/2\right\rangle$	45	(20,20)	0.45	0.08	0.04	0.017	-

The wavelength of the in-plain optical-Lattice in #1,3 is *λ* = 795 nm and in #2,4 is *λ* = 780 nm^121^. In calculating the fidelity, spontaneous emission of *nD* state at 300 K environment temperature is considered^89^.

Further confinement of electron cloud perpendicular to the lattice, allows smaller qubit-dependent lattice-shift *D*_*z*_. This would enhance the Franc-Condone factor and facilitates the qubit rotation on the spin-dependent lattice. Exciting $\vert n{L}_{j},1/2\rangle$ forms a cloud with *L* angular nodes. The two examples of $\vert 64{D}_{5/2},1/2\rangle$ and $\vert 62{G}_{9/2},1/2\rangle$ are plotted in Fig. 5d, e. These states allow operation in small *D*_*z*_ qubit-dependent lattices with significant overlap of two-qubit Wannier states. The drawback in choosing these types of states is the weak strength of the interaction, see Table 1.

#### Realization with circular states

**Fig. 6:**
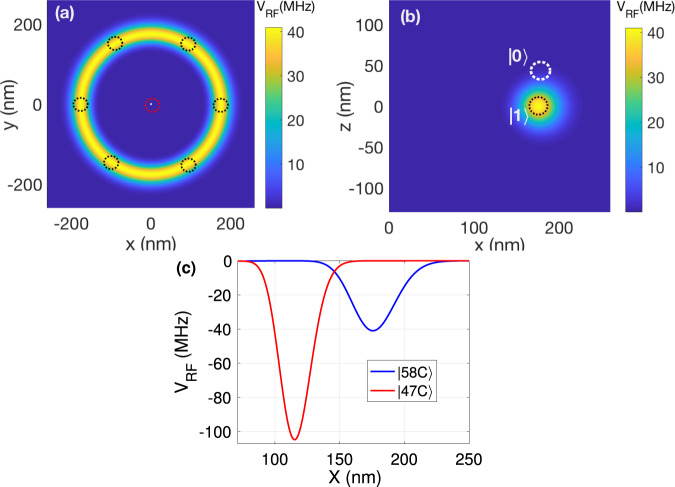
Rydberg-Fermi interaction with circular states. Exciting $\left\vert 58C\right\rangle$, cross-sections of the interaction are plotted along **a** *xy* and **b** *xz*. The white and black circles depict the confining area of the $\left\vert 0\right\rangle$ and $\left\vert 1\right\rangle$ qubit states of the plaquette lattice sites, while the Rb^+^ core is at the origin. **c** The minimal overlap of the interaction profile of $\left\vert 47C\right\rangle$ and $\left\vert 58C\right\rangle$ could be used for laser switching of the circular Rydberg-Fermi interaction, see the text.

The recent advances in fast transition to the Rydberg circular states^110,111^, would make them an ideal choice for the Rydberg-Fermi gates’ application. The ponderomotive potential of focused Laguerre-Gauss (LG) beams, enables site addressing in exciting circular states^111^. Exciting the circular state $\left\vert 58C\right\rangle$ of a ^87^*Rb* atom, the electron would be confined at the position of neighboring qubits at 176 nm, see Fig. 6a, b. Considering a plaquette atom with Gaussian ground motional state of FWHM = 18 nm and qubit-dependent lattice shift of *D* = 50 nm, the interaction would be quantified according to Eq. (2) as

11 $${\bar{V}}_{{{{{{{{\rm{RF}}}}}}}}\left\vert {1}_{l}\right\rangle }=\, 	 39\,{{{{{{{\rm{MHz}}}}}}}}, \quad{{{{{{{{\rm{MD}}}}}}}}}_{{V}_{{{{{{{{\rm{RF}}}}}}}}\left\vert {1}_{l}\right\rangle }}=4\,{{{{{{{\rm{MHz}}}}}}}}\\ {\bar{V}}_{{{{{{{{\rm{RF}}}}}}}}\left\vert {0}_{l}\right\rangle }=\, 	 0.5\,{{{{{{{\rm{MHz}}}}}}}}, \quad{{{{{{{{\rm{MD}}}}}}}}}_{{V}_{{{{{{{{\rm{RF}}}}}}}}\left\vert {0}_{l}\right\rangle }}=0.27\,{{{{{{{\rm{MHz}}}}}}}}$$

The other advantage of the circular state comes from the minimized overlap of the wave-function with the ionic-core which results in an enhanced lifetime in the order of minutes^112^.

In an alternative approach, one can encode the central atom’s $\left\vert {0}_{c}\right\rangle$ and $\left\vert {1}_{c}\right\rangle$ qubit states in the ground $\left\vert g\right\rangle$ and the circular state $\left\vert nC\right\rangle$. The laser transition between close circular states $\left\vert {1}_{c}\right\rangle =\left\vert nC\right\rangle$ and $\left\vert (n+11)C\right\rangle$^113^ could be used to turn on the Ryd-Fermi interaction. The radial interaction profile of $\left\vert 58C\right\rangle$ and $\left\vert 47C\right\rangle$ are plotted in Fig. 6c showing maximum and minimum overlap with a plaquette atom confined at 176 nm. The interaction of $\left\vert {1}_{c}\right\rangle$ central qubits would be compensated with global dynamical decoupling (DD) sequences such as WAHUHA^114^.

#### Strong resonance scattering in Cs atoms

**Fig. 7:**
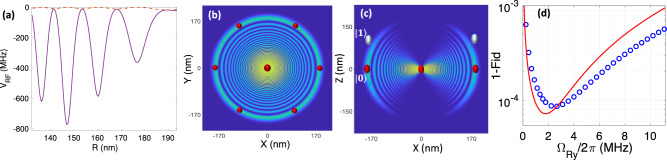
Interaction enhancement by resonance scattering in Cs atoms. **a** PEC with S- and P-wave scattering in Cs atoms. The coupling of the Rydberg state $\vert 46{D}_{5/2},5/2\rangle$ with the neighboring states $\left\vert 43H+42H+47P+48S\right\rangle$ is considered under Eq. (12). Interaction strength is plotted across the radial direction from the Cs^+^ core with *θ* = *π*/2 in spherical coordinates. The solid line and dashed line show the total and exclusive s-wave scattering respectively. **b**, **c** The cross-sections of $\vert 46{D}_{5/2},5/2\rangle$ Rydberg wave-function along **b** *xy* and **c** *xz* are plotted. The red and white circles depict the confining area of the $\left\vert 0\right\rangle$ and $\left\vert 1\right\rangle$ qubit states of a plaquette lattice site, while the Cs^+^ core is at the origin. **d** The infidelity of C_6_-NOT operation as a function of Rydberg exciting Rabi-frequency. The solid line is based on the analytic model for a square laser pulse while the circles are based on the numerical simulation of Eq. (9), obtained for a Gaussian pulse ${{{\Omega }}}_{r}\exp (-\frac{{(t-T/2)}^{2}}{2{\sigma }^{2}})\exp (-\frac{{(T/2)}^{2}}{2{\sigma }^{2}})$ with *σ* = *T*/5 and a pulse duration *T* given by $\int\nolimits_{0}^{T}{{\Omega }}(t){{{{{{{\rm{d}}}}}}}}t=2\pi$. The fidelity quantified by Eq. (10), encounters spontaneous emission and rotation errors.

The interaction-to-loss ratio could also get enhanced by harnessing the p-wave near-resonance scattering from ^133^Cs atoms. Compared to ^87^Rb, this resonance occurs at smaller electron kinetic energies corresponding to larger interatomic distances^62^. Figure 7a plots the potential energy curves (PEC) coupled with the neighboring Rydberg states $\left\vert 43H+42H+47P+48S\right\rangle +\left\vert 6S\right\rangle$ under Ryd-Fermi interaction

12 $${V}_{{{{{{{{\rm{RF}}}}}}}}}=\left(2\pi \frac{\tan ({\delta }^{s})}{k(R)}-6\pi \frac{\tan ({\delta }^{p})}{{k}^{3}(R)}{\overleftarrow{\nabla }}_{{{{{{{{\bf{r}}}}}}}}}.{\overrightarrow{\nabla }}_{{{{{{{{\bf{r}}}}}}}}}\right)\delta ({{{{{{{\bf{r}}}}}}}}-{{{{{{{\bf{R}}}}}}}}).$$

Here $\left\vert nH\right\rangle ={\sum }_{l,m}\left\vert n,l,m\right\rangle$ represents the Hydrogen state encountering semi-degenerate orbital angular momentum numbers 2 < *l* < *n*. The matrix elements in the manifold of coupled states are given by

13 $${H}_{nlm,{n}^{{\prime} }{l}^{{\prime} }{m}^{{\prime} }}({{{{{{{\bf{R}}}}}}}})=\left\langle {\psi }_{nlm}({{{{{{{\bf{R}}}}}}}})\right\vert {V}_{{{{{{{{\rm{RF}}}}}}}}}\left\vert {\psi }_{{n}^{{\prime} }{l}^{{\prime} }{m}^{{\prime} }}({{{{{{{\bf{R}}}}}}}})\right\rangle;\quad {H}_{nlm,nlm}=-\frac{Ry}{{n}^{* 2}}$$

where *Ry* is the Rydberg constant of *Cs* atoms and n^*^ is the effective Rydberg principal number. Diagonalizing 8000 coupled states, the energy potential is plotted in Fig. 7. In a UV optical-lattice with *λ* = 350 nm, the Fermi scattering of Rydberg electron from the neighboring lattice site would result in about 400 MHz level-shift of the Rydberg level ideal for fast quantum operations.

To evaluate the p-wave scattering of Rydberg electron from the neighboring ground state atom, the gradient of the Rydberg wave-function $\psi ={R}_{nl}(r){Y}_{l}^{m}(\theta ,\phi )$ at the position of the neighboring lattice site is required which is

14 $$\nabla \psi (r,\theta ,\phi )=\, 	 \left[\begin{array}{c}\frac{\partial {R}_{nl}}{\partial r}{Y}_{l}^{m}\\ \frac{1}{r}{R}_{nl}\frac{\partial {Y}_{l}^{m}}{\partial \theta }\\ \frac{1}{r\sin \theta }{R}_{nl}\frac{\partial {Y}_{l}^{m}}{\partial \phi }\\ \end{array}\right] \\ =\, 	 \left[\begin{array}{c}\frac{\partial {R}_{nl}(r)}{\partial r}{Y}_{l}^{m}(\theta ,\phi )\\ \frac{\sqrt{{l}^{2}-{m}^{2}}{R}_{nl}(r)}{2r}[{Y}_{l}^{m+1}(\theta ,\phi ){e}^{-{{{{{{{\rm{i}}}}}}}}\phi }-(l+m+1){Y}_{l}^{m-1}(\theta ,\phi ){e}^{{{{{{{{\rm{i}}}}}}}}\phi }]\\ {{{{{{{\rm{i}}}}}}}}m\frac{\psi (r,\theta ,\phi )}{r\sin (\theta )}\\ \end{array}\right]$$

in the spherical coordinate. The radial wave-function and its derivative are calculated numerically using the Numerov technique^115^.

In a triangular lattice of Fig. 7b, the in-plane *x*−*y* Cs trap is formed by 350 nm UV laser dressing the ground state to $\left\vert 10P\right\rangle$ state. Alternative approaches for the implementation of small lattice constants using dual-species lattices and dark-state approach are discussed in^44,116^. Considering the Gaussian ground motional state with a half-width at 1/e maximum of *σ*_*x*,*y*_ = 8.7 nm, the atom would accommodate within a single lobe of *V*_RF_, see Fig. 7b, c. For spin-dependent trap perpendicular to the plane (along the *z*) the *λ* = 870 nm laser could be used for dressing $\left\vert 6S\right\rangle$ to $\left\vert 6P\right\rangle$, with *U*/2*π* = 2 MHz, *σ*_*z*_ = 20 nm, *D*_*z*_ = 100 nm. Exciting the target atom to $\vert 46{D}_{5/2},5/2\rangle$, the Rydberg-Fermi interaction averaged over the l^*th*^ plaquette atom’s wave-function in the ground motional state would be

15 $${\bar{V}}_{{{{{{{{\rm{RF}}}}}}}}\left\vert {1}_{l}\right\rangle }=\, 	 365\,{{{{{{{\rm{MHz}}}}}}}}, \quad{{{{{{{{\rm{MD}}}}}}}}}_{{V}_{{{{{{{{\rm{RF}}}}}}}}\left\vert {1}_{l}\right\rangle }}=0.07\,{{{{{{{\rm{MHz}}}}}}}}\\ {\bar{V}}_{{{{{{{{\rm{RF}}}}}}}}\left\vert {0}_{l}\right\rangle }=\, 	 2\,{{{{{{{\rm{MHz}}}}}}}}, \quad{{{{{{{{\rm{MD}}}}}}}}}_{{V}_{{{{{{{{\rm{RF}}}}}}}}\left\vert {0}_{l}\right\rangle }}=0.06\,{{{{{{{\rm{MHz}}}}}}}}$$

see Eq. (2) for the definitions. This contrast of spin-dependent level-shift with narrow lines allows fast selective laser excitation of the central atom conditioned on the plaquette qubits’ configurations. This would result in high fidelity operation of C_6_-NOT gate as depicted in Fig. 7d. The fidelity is quantified along the same lines described in Fig. 4.

### Qubit-Rotation in the spin-dependent lattice

**Fig. 8:**
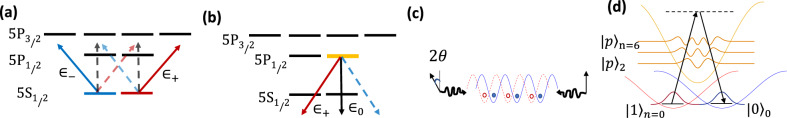
Qubit rotation in the spin-dependent lattice. **a** In Rb (Cs), tuning the trapping laser between 5*P*_3/2_ (6*P*_3/2_) and 5*P*_1/2_ (6*P*_1/2_), the polarizability of qubit states $\left\vert 0\right\rangle$ and $\left\vert 1\right\rangle$ are given by left *ε*_−_ and right *ε*_+_ circularly polarized lights respectively. **b** The same fields, trap the $\left\vert p\right\rangle =\vert 5(6){P}_{1/2},1/2\rangle$ electronic state with *ε*_0_ and *ε*_+_ elements. **c** The relative polarization of 2*θ* between counter-propagating linearly polarized lights could be tuned to trap the intermediate state between the qubit states (**d**). The Raman transition in a dual spin/spatial encoded qubit would be modified by the Frank-Condon factor.

A spin-dependent lattice provides dual spin/spatial encoding of the qubit. A Raman transition coherently transfers an atom from one internal state to the other, thereby causing hopping between the two Wannier-functions^76–78^, see Fig. 8d. The polarizability of the qubit states $\left\vert 0\right\rangle$, $\left\vert 1\right\rangle$ and the intermediate electronic-level $\vert 5{P}_{1/2},1/2\rangle$ in the optical lattice are given by different light polarization elements, see Fig. 8a,b. Hence by tuning the polarization angle *θ* between counter-propagating linearly polarized lights it is possible to confine the intermediate state between the two-qubit-dependent lattices, see Fig. 8c, d.

Here the Raman-assisted transition of a trapped neutral atom between the two spin-dependent lattices centered at *l*_0_ and *l*_1_ is analyzed. A Raman transition between the atom’s internal states $\left\vert 0\right\rangle$ and $\left\vert 1\right\rangle$ makes the atom experience a different trap shifted by *D* where the initial and final vibrational wave-functions have overlap. This scheme resembles the Franck-Condon principle in molecular physics. Under the Born-Oppenheimer approximation, the electronic and the nuclear motions are separated and hence the effective wave-function would be presented as a product of the electronic wave-function and the vibrational wave-function

16 $${\psi }_{{l}_{i}}({{{{{{{\bf{R}}}}}}}},{{{{{{{\bf{r}}}}}}}})=w({{{{{{{\bf{R}}}}}}}}-{{{{{{{{\bf{l}}}}}}}}}_{i}){\psi }_{e,i}({{{{{{{\bf{R}}}}}}}},{{{{{{{\bf{r}}}}}}}})$$

where **r** and **R** are addressing the electronic and center of mass positions. The Wannier function of the *l*th site in the *i* ∈ {0, 1, *p*} spin dependent lattices is given by *w*(**R** − **l**_*i*_). The electric dipole transition from a state A to an excited state B is given by

17 $$\left\langle {\psi }_{A}\right\vert ({{{{{{{\bf{R}}}}}}}}+{{{{{{{\bf{r}}}}}}}})\left\vert {\psi }_{B}\right\rangle =\left\langle w({{{{{{{\bf{R}}}}}}}}-{{{{{{{{\bf{l}}}}}}}}}_{A})| w({{{{{{{\bf{R}}}}}}}}-{{{{{{{{\bf{l}}}}}}}}}_{B})\right\rangle \langle {\psi }_{e,A}\vert {{{{{{{\bf{r}}}}}}}}\vert {\psi }_{e,B}\rangle .$$

Here we have the orthogonality of the electronic eigenstates but the vibrational states are belonging to different traps and do not need to be orthogonal. Also using the Condon approximation, the dipole transition of electronic states is assumed independent of nuclear coordinates. In conclusion, the dipole transition would be modified by the overlap of the wave-functions i.e. Franck-Condon factor $f=\left\langle w({{{{{{{\bf{R}}}}}}}}-{{{{{{{{\bf{l}}}}}}}}}_{A})| w({{{{{{{\bf{R}}}}}}}}-{{{{{{{{\bf{l}}}}}}}}}_{B})\right\rangle$.

#### Effective qubit-rotation rate

The Hamiltonian *H* = *H*_0_ + *H*_*d*_ consists of the energy level of electronic states *H*_0_, and the dipole transitions *H*_*d*_. The Hamiltonian of the system in the rotating wave approximation and in the basis $\{{\left\vert 0\right\rangle }_{0},{\left\vert 1\right\rangle }_{0},{\left\vert p\right\rangle }_{0},{\left\vert p\right\rangle }_{1},{\left\vert p\right\rangle }_{2},...,{\left\vert p\right\rangle }_{n}\}$ would be

18 $$\tilde{H}=\left[\begin{array}{ccccc}0&0&{f}_{0{p}_{0}}{{{\Omega }}}_{0}/2&\ldots \,&{f}_{0{p}_{n}}{{{\Omega }}}_{0}/2\\ 0&\delta &{f}_{1{p}_{0}}{{{\Omega }}}_{1}/2&\ldots \,&{f}_{1{p}_{n}}{{{\Omega }}}_{1}/2\\ {f}_{0{p}_{0}}{{{\Omega }}}_{0}/2&{f}_{1{p}_{0}}{{{\Omega }}}_{1}/2&{{\Delta }}&\ldots \,&0\\ \vdots &\vdots &\vdots &\ddots &\vdots \\ {f}_{0{p}_{n}}{{{\Omega }}}_{0}/2&{f}_{1{p}_{n}}{{{\Omega }}}_{1}/2&0&\ldots \,&{{\Delta }}-n{\omega }_{tr}\\ \end{array}\right]$$

where Δ = *ω*_*L*0_ − (*ω*_*p*0_ − *ω*_0_) = *ω*_*L*1_ − (*ω*_*p*0_ − *ω*_1_), *δ* = (*ω*_*L*0_ − *ω*_*L*1_) − (*ω*_1_ − *ω*_0_) being the one and two-photon detunings and subscript *L* distinguishes the laser frequency from atomic energies. The Frank-Condon factors ${f}_{in}=\int{w}_{i}^{* }({{{{{{{\bf{x}}}}}}}}-{{{{{{{\bf{{l}}}}}}}_{i}}}){w}_{{p}_{n}}({{{{{{{\bf{x}}}}}}}}-{{{{{{{\bf{{l}}}}}}}_{p}}}){{{{{{{\rm{d}}}}}}}}{{{{{{{\bf{x}}}}}}}}$ quantify the overlapping of the qubit states $i={\left\vert 0,1\right\rangle }_{n = 0}$ and intermediate ${\left\vert p\right\rangle }_{n}$ state’s Wannier function ${w}_{{p}_{n}}$ in the *n*th motional state. The Schrödinger equation in the interaction picture could be written in terms of coupled equations:

19 $$\frac{{{{{{{{\rm{d}}}}}}}}{C}_{0}}{{{{{{{{\rm{d}}}}}}}}t} =\, 	 {{{{{{{\rm{i}}}}}}}}\mathop{\sum }\limits_{j=0}^{n}\frac{{f}_{0j}{{{\Omega }}}_{0}}{2}{C}_{{p}_{j}}\\ \frac{{{{{{{{\rm{d}}}}}}}}{C}_{1}}{{{{{{{{\rm{d}}}}}}}}t} =\, 	 {{{{{{{\rm{i}}}}}}}}\mathop{\sum }\limits_{j=0}^{n}\frac{{f}_{1j}{{{\Omega }}}_{1}}{2}{C}_{{p}_{j}}\\ \frac{{{{{{{{\rm{d}}}}}}}}{C}_{{p}_{j}}}{{{{{{{{\rm{d}}}}}}}}t} =\, 	 {{{{{{{\rm{i}}}}}}}}\left(\frac{{f}_{0j}{{{\Omega }}}_{0}}{2}{C}_{0}+\frac{{f}_{1j}{{{\Omega }}}_{1}}{2}{C}_{1}+[{{\Delta }}-j{\omega }_{tr}]{C}_{{p}_{j}}\right)$$

Under the condition that Δ is the dominant term, the intermediate levels could be adiabatically eliminated. The effective Rabi frequency in the two-level system would be

20 $$\tilde{{{\Omega }}}=\frac{{{{\Omega }}}_{0}{{{\Omega }}}_{1}}{4{{\Delta }}}\mathop{\sum }\limits_{j=0}^{n}{f}_{0j}{f}_{j1}$$

and the effective detuning of the two-level system would be

21 $${\delta }_{{{{{{{{\rm{eff}}}}}}}}}=\delta -\mathop{\sum }\limits_{j=0}^{n}\frac{{f}_{1j}^{2}{{{\Omega }}}_{1}^{2}-{f}_{0j}^{2}{{{\Omega }}}_{0}^{2}}{4{{\Delta }}}$$

The qubit-rotation is performed in the regime of $\tilde{{{\Omega }}}\ll {\omega }_{{{{{{{{\rm{tr}}}}}}}}}$ to avoid exciting the motional Bloch bands.

### Gate simulation under large bandwidth driving pulses

The other concern about pulse duration is related to the pulse bandwidth. While fast operation makes short pulses desirable, short pulses would be wide in bandwidth and might excite the neighboring Rydberg levels^117^ in fan-out or disturb the blockade in Toffoli gate. For the chosen Rydberg levels $\vert 65{P}_{3/2}\rangle$, $\left\vert 64D\right\rangle$ and $\left\vert 75D\right\rangle$ in Fig. 1 and 2, the level spacing to the next dipole accessible Rydberg level would be 17 GHz, 21 GHz and 6 GHz respectively. The fan-out gate operates with stronger Rabi frequencies compared to Toffoli and in principle could operate with shorter pulses *τ* ≳ 140ns. Corresponding pulse bandwidths would be at least three orders of magnitude smaller than the level spacing in the above-mentioned cases.

In the fast operation of the Toffoli gate, the laser pulse bandwidth might be comparable with the interaction-induced level-shift suppressing the blockade at the heart of the scheme. Circle signs in Fig. 4 quantify the gate’s operation by simulating the master equation encountering the pulse bandwidth, the spontaneous emissions in two-photon excitation, and the de-phasing terms associated with laser line-widths as described below. The effective Rabi frequency in two-photon excitation ${{{\Omega }}}_{r}=\frac{{{{\Omega }}}_{420}{{{\Omega }}}_{1013}}{2{\delta }_{p}}$ is obtained by Ω_420_ = Ω_1013_ lasers that are detuned from intermediate $\left\vert p\right\rangle$ level by *δ*_*p*_/2*π* = 5 GHz. The Gaussian pulses ${{{\Omega }}}_{r}{e}^{-\frac{{(t-T/2)}^{2}}{2{\sigma }^{2}}}{e}^{-\frac{{(T/2)}^{2}}{2{\sigma }^{2}}}$ are considered in the numerics with *σ* = *T*/5 and a pulse duration *T* given by $\int\nolimits_{0}^{T}{{\Omega }}(t){{{{{{{\rm{d}}}}}}}}t=2\pi$. The driving Hamiltonian of Eq. (9) is a function of the pulse frequency elements *ω*_*l*2_ and *ω*_*l*1_ both in Rabi frequencies and detunings. Over the two-photon excitation, the Fourier transform of the two laser pulses would be ${{{\Omega }}}_{1}({\omega }_{l1})={{{\Omega }}}_{420}{e}^{-\frac{{\omega }_{l1}-{\omega }_{1c}}{4{w}^{2}}}$ and ${{{\Omega }}}_{2}({\omega }_{l2})={{{\Omega }}}_{1013}{e}^{-\frac{{\omega }_{l2}-{\omega }_{2c}}{4{w}^{2}}}$ with *ω*_*c*_ indicating the central frequency of the pulse and the pulse bandwidth is presented by *w* = 1/*σ*. The laser detunings from the intermediate and Rydberg levels *δ*_*p*_ = *ω*_*l*1_ − *ω*_1*p*_ and Δ = *ω*_*l*1_ + *ω*_*l*2_ − *ω*_1*r*_ are also a function of the pulse frequency elements. In Fig. 4a,b the master equation (Eq. (22)) is simulated for distinct pulse frequencies *ω*_*l*1_, *ω*_*l*2_ and the final results are averaged over the Gaussian frequency profile of the two laser pulses.

In the operation, the target atom is subject to de-phasing and decay terms that are encountered by the master equation

22 $${\partial }_{t}\hat{\rho }=-{{{{{{{\rm{i}}}}}}}}[\hat{H},\hat{\rho }]+{{{{{{{\mathcal{L}}}}}}}}(\hat{\rho })$$

where the Liouvillian term ${{{{{{{\mathcal{L}}}}}}}}(\rho )={\sum}_{\beta }{{{{{{{\mathcal{D}}}}}}}}({c}_{\beta })\rho$ with ${{{{{{{\mathcal{D}}}}}}}}(c)\rho =c\rho {c}^{{{{\dagger}}} }-1/2({c}^{{{{\dagger}}} }c\rho +\rho {c}^{{{{\dagger}}} }c)$ in the Lindblad form governs the dissipative time evolution. Lindblad terms encounter spontaneous emission from the intermediate level to the qubit states ${c}_{1p}=\sqrt{{\gamma }_{p}/2}\left\vert 1\right\rangle \left\langle p\right\vert$, ${c}_{0p}=\sqrt{{\gamma }_{p}/2}\left\vert 0\right\rangle \left\langle p\right\vert$ as well as the loss of population from Rydberg state to other electronic states ${c}_{or}=\sqrt{{\gamma }_{r}}\left\vert o\right\rangle \left\langle r\right\vert$. Furthermore, the de-phasing terms associated by the lasers’ linewidth are included as ${c}_{11}=\sqrt{{{{\Gamma }}}_{11}}\left\vert 1\right\rangle \left\langle 1\right\vert$, ${c}_{pp}=\sqrt{{{{\Gamma }}}_{pp}}\left\vert p\right\rangle \left\langle p\right\vert$, ${c}_{rr}=\sqrt{{{{\Gamma }}}_{rr}}\left\vert r\right\rangle \left\langle r\right\vert$, where Γ_11_ = (*γ*_Lock_ + *γ*_*l*1_ − *γ*_*l*2_)/2, Γ_*pp*_ = (−*γ*_Lock_ + *γ*_*l*1_ + *γ*_*l*2_)/2, Γ_*rr*_ = (*γ*_Lock_ − *γ*_*l*1_ + *γ*_*l*2_)/2^118^ with *γ*_*l*1_ and *γ*_*l*2_ being the line-widths of Ω_1_ and Ω_2_ lasers (corresponding to 420 nm and 1013 nm lasers respectively). The coherence in two-photon excitation would be sensitive to the linewidth of the Lock *γ*_Lock_ when the lasers are locked out of phase^118^, which could be suppressed to less than 1 kHz^33^.

### Single site addressing

#### Applying local light-shift

The Laser cross-talk and misalignment could affect the accuracy of gate operation in compact lattices. The population that does not return to the qubit basis over the Rydberg excitation would be considered as loss. Inspired by^13^, single-site addressing could be performed by applying site-selective differential light-shift to the $\left\vert 1\right\rangle \left\langle r\right\vert$ transition. Focusing the 788 nm auxiliary laser on the targeted site, only the desired atom would get in-resonance with the Rydberg exciting laser.

**Fig. 9:**
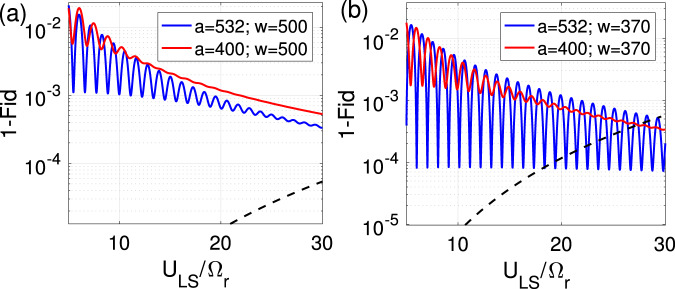
Single-site addressing—effects of laser cross-talk and misalignment on gate’s fidelity. The population that does not return to the qubit basis after gate operation would be considered as loss. The errors are averaged over the laser’s misalignment area which is a circle with radius *r*_0_ and also over the Wannier state of atoms. The reported error is averaged over all qubit configurations in a C_4_-NOT gate. Red and blue lines are corresponding to systems described in Fig. 1 and 2 with lattice constants *a* = 400 and 532 nm. The solid and dashed lines are corresponding to errors of plaquette ${\bar{E}}_{p}$ and central atoms ${\bar{E}}_{c}$. To apply the single-site addressing the 788 nm laser is focused to 1/*e*^2^ intensity waist of **a** *w* = 500 nm and **b** *w* = 370 nm with an alignment accuracy of *r*_0_ = 25 nm, generating a differential light-shift *U*_LS_ on $\left\vert 1\right\rangle \left\langle r\right\vert$ transition. The Oscillation is due to the change of effective Rabi frequency $\tilde{{{\Omega }}}$, which leads to different values of Rydberg leakage at the plaquette sites after the gate operation.

To quantify the single site addressing efficiency, we consider a microscope with NA = 0.68 that focuses the 788 nm light to 1/*e*^2^ intensity waists of *w* = 370 nm and 500 nm^13^. The alignment accuracy of 25 nm has been achieved for single-site addressing^13^, which is subject to improvement by e.g., sub-wavelength localization of atoms^10,11^. The generated light-shift by the focused laser has the form of ${U}_{{{{{{{{\rm{LS}}}}}}}}}(x,y)={U}_{{{{{{{{\rm{LS}}}}}}}}}{{{{{{{{\rm{e}}}}}}}}}^{-2({(x-{x}_{0})}^{2}+{(y-{y}_{0})}^{2})/{w}^{2}}$ with ${r}_{0}=\sqrt{{x}_{0}^{2}+{y}_{0}^{2}}$ being the laser misalignment. At the central site, this misalignment would cause a detuning ${{\Delta }}({r}_{0})={U}_{{{{{{{{\rm{LS}}}}}}}}}(1-{e}^{-2{r}_{0}^{2}/{w}_{0}^{2}})$ in $\left\vert 1\right\rangle \left\langle r\right\vert$ transition that changes the effective Rabi frequency $\tilde{{{\Omega }}}=\sqrt{{{{\Omega }}}^{2}+{{{\Delta }}}^{2}}$. Hence for the central atom, starting at $\left\vert 1\right\rangle$ qubit state, after the gate operation time $\tau =\frac{2\pi }{{{\Omega }}}$, the qubit state would not be fully retrieved, which results in an error of ${E}_{c}=\frac{{{{\Omega }}}^{2}}{{\tilde{{{\Omega }}}}^{2}}{\sin }^{2}(\tilde{{{\Omega }}}\tau /2)$. Considering the uncertainty in addressing a specific site within distance *r*_0_, the error of the centered atom must be averaged ${\bar{E}}_{c}=\frac{1}{\pi {r}_{0}^{2}}\int_{0}^{{r}_{0}}{E}_{c}(r)2\pi r\,{{{{{{{\rm{d}}}}}}}}r$. Taking into account the error distribution profile, the optimum operation time would be modified from $\tau =\frac{2\pi }{{{\Omega }}}$ to ${\tau }_{{{{{{{{\rm{opt}}}}}}}}}=\frac{2\pi }{\sqrt{{{{\Omega }}}^{2}+{{\Delta }}(\frac{3}{4}{r}_{0})}}$. The averaged error of the central atom ${\bar{E}}_{c}$ is plotted by dashed lines in Fig. 9 as a function of *U*_*LS*_/Ω. The infidelities caused by laser misalignment could be suppressed by the spatial beam shaping^119^.

Concerning the laser cross-talk, the detuning experienced by the neighboring plaquette atoms must be large enough to avoid population leakage out of the qubit basis. The leakage probability of a plaquette atom in $\left\vert 1\right\rangle$ qubit state over the operation time *τ*_opt_ is given by ${E}_{p}=\frac{{{{\Omega }}}^{2}}{{{{\Omega }}}^{2}+{{\Delta }}{(| {{{{{{{\bf{r}}}}}}}}| )}^{2}}{\sin }^{2}(\frac{{{{\Omega }}}^{2}+{{\Delta }}{(| {{{{{{{\bf{r}}}}}}}}| )}^{2}}{{{{\Omega }}}^{2}+{{\Delta }}{(\frac{3}{4}{r}_{0})}^{2}}\pi )$ where ∣**r**∣ = ∣**a** − **r**_**l**_ − **r**_**p**_∣ is the distance from the center of the laser beam to the neighboring plaquette atom. Here **a** is the distance vector between centered and a plaquette site, and one needs to average over the laser misalignment **r**_**l**_ and also over the position of the plaquette atom **r**_**p**_ considering its Wannier wave-function to find the average loss of a plaquette atom ${\bar{E}}_{p}$ in $\left\vert 1\right\rangle$ state. The variation of the averaged plaquette error is plotted by solid lines in Fig. 9a, b as a function of *U*_LS_/Ω. The Oscillation is due to the change of effective Rabi frequency $\tilde{{{\Omega }}}$, which leads to different values of Rydberg leakage at the plaquette sites after the gate operation. At large *U*_*LS*_/Ω and also for weak laser focusing (large *w*) the variation of detuning over the plaquette atoms’ wave-functions would be large and hence averaging the error over **r**_1_ and **r**_*p*_ washes the oscillation pattern. The laser cross-talk could be suppressed by using two species lattices^44,90–92^. Considering both plaquette error ${\bar{E}}_{p}$ and the central atom error ${\bar{E}}_{c}$, Fig. 9 shows that single-site operations with high fidelity is achievable in the designed setups discussed in the main text.

The scattering of the auxiliary laser could also affect the fidelity. To give an example a 420 nm auxiliary laser focused to 370 nm, dresses the $\left\vert 1\right\rangle$ qubit state by $\vert 6{P}_{1/2}\rangle$ with Ω_*LS*_/2*π* = 200 MHz and the laser detuning of Δ_*LS*_/Ω_*LS*_ = − 150. This laser imposes a differential light-shift of ${U}_{LS}={{{\Omega }}}_{LS}^{2}/4{{{\Delta }}}_{LS}=-2.1$ MHz on the $\left\vert 1\right\rangle -\left\vert r\right\rangle$ transition. In a Toffoli gate with Ω_*r*_/2*π* = 30 kHz (Fig. 4), the single-site addressing infidelity of 0.003 is expected, see Fig. 9b. Over the 30 μs operation time, the photon scattering from the $\vert 6{P}_{1/2}\rangle$ state would cause 0.0015 gate infidelity. An alternative approach is to initially change the hyperfine state of the desired site^13^ and then excite the new auxiliary hyperfine state to the Rydberg level. In this case, it would be important that both hyperfine states get trapped at the same position in the qubit-dependent lattice of Fig. 1a–c. A possible choice is changing $\left\vert 0\right\rangle =\vert F=1,{m}_{f}=1\rangle$ to $\vert F=2,{m}_{f}=-1\rangle$ which has the same distribution of *m*_*j*_ components and hence experiences the same trapping potential.

#### Interferometric approach

**Fig. 10:**
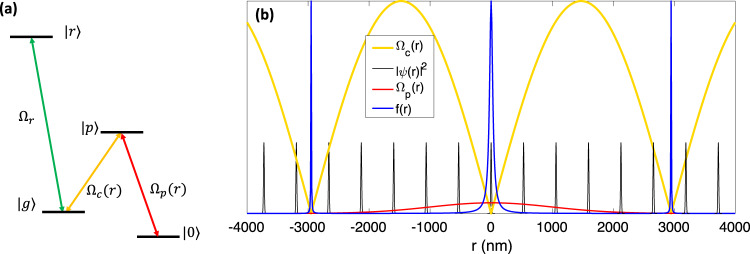
Dark-state single site addressing technique. **a** the level scheme containes a Λ configuration transferring the population from $\left\vert 0\right\rangle$ to $\left\vert g\right\rangle$ at the nodes of Ω_*c*_ standing-wave where Ω_*c*_ ≪ Ω_*p*_. The transferred atom would then get excited to the Rydberg state. **b** The spatial profile of the transition Rabi frequencies Ω_*c*,*p*_ as well as the $\left\vert 0\right\rangle \left\langle g\right\vert$ transition probabilities *f*(*x*) and the wave-function density ∣*ψ*(*x*)∣^2^ of the atoms in $\left\vert 0\right\rangle$ qubit states are plotted. *f*(*r*) maps the ∣*ψ*(*x*)∣^2^ to $\left\vert g\right\rangle$ state upon the spatial overlap which is designed to only occurs at the targeted site. **b** Sample applied parameters are Ω_*c*_/Ω_*p*_ = 30, $\tilde{k}=1 \, {{\mu}} {m}^{-1}$ and the Gaussian width of Ω_*p*_ laser is *w* = 2 μm.

Single-site addressing could be realized with precisions below the diffraction limit using an interferometer technique introduced before in sub-wavelength localization^8–12^. Over this process the qubit state $\left\vert 0\right\rangle =\vert 5S,F=1,{m}_{f}=1\rangle$ of the desired site would be changed to an auxiliary hyperfine state $\vert g\rangle =\vert 5S,F=2,{m}_{f}=-1\rangle$ via an intermediate level $\left\vert 6P\right\rangle$. The three-level Λ transition is operated by a standing-wave driving field (Ω_*c*_) and a focused laser (Ω_*p*_), see Fig. 10a, b. The standing-wave is formed in each dimension by counter-propagating fields ${{{\Omega }}}_{c1q}\exp (ikq)$ and ${{{\Omega }}}_{c2q}\exp (-ikq+{\phi }_{q})$ where *q* ∈ {*x*, *y*}. The transition occurs under the dark state STIRAP mechanism. The dark-state in the described Λ system is a superposition of the $\left\vert g\right\rangle$ and $\left\vert 0\right\rangle$ states with spatially varying amplitudes:

23 $$\left\vert D({{{{{{{\bf{r}}}}}}}})\right\rangle =\frac{1}{\sqrt{{{{\Omega }}}_{c}{({{{{{{{\bf{r}}}}}}}})}^{2}+{{{\Omega }}}_{p}{({{{{{{{\bf{r}}}}}}}})}^{2}}}[{{{\Omega }}}_{c}({{{{{{{\bf{r}}}}}}}})\left\vert 0\right\rangle -{{{\Omega }}}_{p}({{{{{{{\bf{r}}}}}}}})\left\vert g\right\rangle ].$$

To apply the transition, first the Ω_*c*1*q*_ field would be applied. The probe field would then be applied focused on the targeted site with a Gaussian profile ${{{\Omega }}}_{p}({{{{{{{\bf{r}}}}}}}})={{{\Omega }}}_{p}{{{{{{{{\rm{e}}}}}}}}}^{-{({{{{{{{\bf{r}}}}}}}}-{{{{{{{{\bf{r}}}}}}}}}_{{{{{{{{\bf{0}}}}}}}}})}^{2}/{w}^{2}}$ and Ω_*p*_ ≪ Ω_*c*_. In the next step Ω_*c*2*q*_ would be applied adiabatically^11^ to form the standing-wave with a node being adjusted on the position of the targeted site via the *ϕ*_2*q*_ angle. At the nodes of Ω_*c*_ standing-wave Ω_*p*_(*r*) ≫ Ω_*c*_(*r*) the dark-state composition is predominantly $\left\vert g\right\rangle$ while away from the nodes Ω_*p*_(*r*) ≪ Ω_*c*_(*r*) the dark state would remain at $\left\vert 0\right\rangle$ qubit state.

Considering the wave-function density ∣*ψ*(**r**)∣^2^ of atoms initialized in $\left\vert 0\right\rangle$ states, the local population of $\left\vert g\right\rangle$ state after applying Ω_*p*,*c*_ fields would be *f*(**r**)∣*ψ*(**r**)∣^2^ where *f*(**r**) is obtained from Eq. (23) as

24 $$f(r)=\frac{{{{\Omega }}}_{p}^{2}{{{{{{{{\rm{e}}}}}}}}}^{-\frac{2{({{{{{{{\bf{r}}}}}}}}-{{{{{{{{\bf{r}}}}}}}}}_{{{{{{{{\bf{0}}}}}}}}})}^{2}}{{w}^{2}}}}{{{{\Omega }}}_{p}^{2}{{{{{{{{\rm{e}}}}}}}}}^{-\frac{2{({{{{{{{\bf{r}}}}}}}}-{{{{{{{{\bf{r}}}}}}}}}_{{{{{{{{\bf{0}}}}}}}}})}^{2}}{{w}^{2}}}+{{{\Omega }}}_{c}^{2}{\sin }^{2}\tilde{k}(x-{x}_{0}){\sin }^{2}\tilde{k}(y-{y}_{0})},$$

where $\tilde{k}=k\sin \theta /2$ with *θ* being the angle between the Ω_*c*1_ and Ω_*c*2_ lasers. Figure 10b plots the narrow peaks of *f*(*r*) at the nodes of Ω_*c*_. Going away from the focusing point of Ω_*p*_ at **r**_**0**_, the profile width of *f*(*r*) gets narrower and disappears. The full width at half maximum of an *f* peak located at ${r}^{{\prime} }$ would be given by ${{{{{{{{\rm{FWHM}}}}}}}}}_{f({r}^{{\prime} })}=2{{{\Omega }}}_{p}\exp (-{({{{{{{{{\bf{r}}}}}}}}}^{{\prime} }-{{{{{{{{\bf{r}}}}}}}}}_{{{{{{{{\bf{0}}}}}}}}})}^{2}/{w}^{2})/\tilde{k}{{{\Omega }}}_{c}$^10,11^. While the nearest peaks of *f* shown in Fig. 10b do not overlap with the atomic lattice sites the next nearest neighbors are at the position where the amplitude of Ω_*p*_ would approach zero. In the next step, the Ω_*p*_ and Ω_*c*_ lasers would be turned off simultaneously keeping the ratio of Ω_*c*_(*t*)/Ω_*p*_(*t*) constant to preserve the dark state components. At this stage, only the desired site would be in the $\left\vert g\right\rangle$ state and hence would get excited to the Rydberg level by the subsequent Ω_*r*_ laser. Considering the qubit-dependent lattice of Fig. 1a–c, the auxiliary state $\left\vert g\right\rangle$ would experience the same trapping potential as $\left\vert 0\right\rangle$ state since the distribution of *m*_*j*_ components in the two hyperfine states are the same.

The initial calibration of the Ω_*c*_ standing wave with the optical lattice could be done by fluorescence imaging with the approach of ref. ^11^. The nodes of the Ω_*c*_ standing wave could be then moved by high resolution adjusting of the Ω_*c*2*q*_ phase^120^. For the chosen parameters of Fig. 10b applied in a lattice of *a* = 532 nm with the atom confinement of FWHM = 20 nm, and focusing Ω_*p*_ laser to the Gaussian width of 2 μm, the single site addressing infidelity averaged over the qubit configurations would be 0.01. This calculation encounters the population leakage of the neighboring lattices as well as the imperfect transition of the targeted site. Using the two species lattice^44,56,90–92^ could improve the addressing fidelity.

### Implementing stabilizer phase-gate using parallelized gate

The implementation of the stabilizer operator ${B}_{p}=\mathop{\prod}_{i\in p}{\sigma }_{z}^{(i)}$ over the plaquette spins applies in three steps i.e. *B*_*p*_ = *H*^−1^*U*_*g*_*H* where $H=\mathop{\sum}\limits_{i\in \{p,c\}}\exp (i\pi /2{\sigma }_{x}^{(i)})$ is Hadamard applying over all the plaquette and control atoms and *U*_*g*_ is the parallelized Ryd-Fermi gate of Eq. (7). For the control qubit prepared in ${\left\vert 0\right\rangle }_{c}$, the gate *B*_*p*_ coherently transfers the control qubit into the state ${\left\vert 1\right\rangle }_{c}$ (${\left\vert 0\right\rangle }_{c}$) for the odd (even) parity of plaquette spin. The ${A}_{p}={\prod}_{i\in p}{\sigma }_{x}^{(i)}$ stabilizer would be obtained by exclusive application of Hadamard on the control atom. The desired stabilizer-phase gate would be implemented by application of a phase shift on the control qubit, sandwiched by applying/reverting the stabilizer operator *B*_*p*_

25 $${U}_{\square }(\theta )={e}^{i\theta {B}_{p}}={B}_{p}^{-1}{e}^{i\theta {\sigma }_{z}^{(c)}}{B}_{p},$$

with *θ* being optimized between [0, *π*] in QAOA. At the end, the control atom would be factored out by transferring to the ground state.

### Supplementary information

Supplementary Information

## Data Availability

All data needed to evaluate the conclusions in the article are presented in the article and the Supplementary. Additional data related to this paper may be requested from the corresponding author.

## Code availability

Codes could be provided upon reasonable request.
