# Supplementary material for: Scalable quantum processors empowered by the Fermi scattering of Rydberg electrons
Source: Commun Phys. 2023 Mar 31;6(1):57. doi: 10.1038/s42005-023-01174-4 (PMC11041703; doi:10.1038/s42005-023-01174-4)
Supplement: Supplementary file 1 — Supplementary Information [file 42005_2023_1174_MOESM1_ESM.pdf]

Supplemental Materials for "Scalable quantum processors empowered by the Fermi scattering of Rydberg electrons"

Mohammadsadegh Khazali
Institute for Quantum Optics and Quantum Information of the Austrian Academy of Sciences, A-6020 Innsbruck, Austria
School of Physics, Institute for Research in Fundamental Sciences (IPM), Tehran 19395-5531, Iran
Department of Physics, University of Tehran, 14395-547, Tehran, Iran and
mskhazali@yahoo.com

Wolfgang Lechner
Institute for Theoretical Physics, University of Innsbruck, A-6020 Innsbruck, Austria and
Parity Quantum Computing GmbH, A-6020 Innsbruck, Austria

SUPPLEMENTARY NOTE 1: ALTERNATIVE ENCODING OF THE QUBIT STATES

While the chosen qubit states have been widely used in the quantum information experiments in spin-dependent and -independent lattices [1–3], the dual encoding of the qubit could also be realized in other hyperfine states with longer coherence times. One example is the Hadamard combinations of the long-lived hyperfine states $|F=1, m_f=0\rangle$ and $|F=2, m_f=0\rangle$:

$$\begin{aligned}|0\rangle &= (|F=2, m_f=0\rangle - |F=1, m_f=0\rangle)/\sqrt{2} \\ &= |I=3/2, m_I=1/2\rangle |J=1/2, m_j=-1/2\rangle\end{aligned} \quad \text{(S1)}$$

$$\begin{aligned}|1\rangle &= (|F=2, m_f=0\rangle + |F=1, m_f=0\rangle)/\sqrt{2} \\ &= |I=3/2, m_I=-1/2\rangle |J=1/2, m_j=1/2\rangle\end{aligned}$$

In this arrangement the qubit states $|0\rangle$ and $|1\rangle$ would exclusively contain $m_j=-1/2$ and $m_j=1/2$ respectively. Hence they would get trapped by different polarizations of the qubit-dependent lattice as discussed in Fig. 1a-c of the main text. In a Rydberg two-photon excitation with left circularly polarized lights ϵ^- that are red detuned from the $|6P_{1/2}\rangle$ intermediate state, only the $|1\rangle$ state would get excited to the Rydberg level as discussed below. Dipole transitions between the hyperfine states are given by

$$\begin{aligned}&\langle n'l'j'; F'M'|\vec{r}|nlj; FM\rangle = (-1)^{1+l'+s+J+J'+I-M'} \\ &\sqrt{\max(l.l')}\sqrt{(2J+1)(2J'+1)(2F+1)(2F'+1)} \\ &\left\{\begin{matrix} l' & J' & s \\ J & l & 1\end{matrix}\right\}\left\{\begin{matrix} J' & F' & I' \\ F & J & 1\end{matrix}\right\}\left(\begin{matrix} F & 1 & F' \\ M & q & -M'\end{matrix}\right)\langle n'l'|r|nl\rangle\end{aligned} \quad \text{(S2)}$$

where $q=0,\pm1$ for the linear ϵ^0 and $\epsilon^\pm$ circular polarizations of the exciting light. Under the ϵ^- circularly polarized laser $\langle 6P_{1/2}; 1, -1|\vec{r}|5S_{1/2}; 1, 0\rangle = \langle 6P_{1/2}; 1, -1|\vec{r}|5S_{1/2}; 2, 0\rangle$, hence the dipole transition from the $|0\rangle$ ($|1\rangle$) qubit states of Eq. S1 to the $|6P_{1/2}; F=1, M=-1\rangle$ intermediate state would be forbidden (allowed) due to destructive (constructive) interference, see Fig. S1a.

In the upper transition of Fig. S1a, a two-color transition excite a superposition of the Rydberg levels

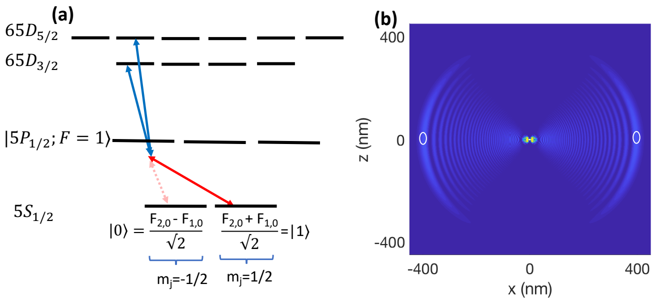

FIG. S1. Qubit encoding in the Hadamard combination of long-lived hyperfine states $|F=2, m_f=0\rangle$ and $|F=1, m_f=0\rangle$, see Eq. S1. The two-qubit states have distinct m_j components allowing qubit-dependent trapping, see Fig. 1a-c of the main text. (a) Applying a two-photon Rydberg excitation via left circularly polarized lights ϵ^- that are red detuned from the $|6P_{1/2}; F=1, m_f=-1\rangle$ intermediate state, only the $|1\rangle$ qubit state would get excited to the Rydberg level, see the main text. The space-dependent Rydberg-Fermi interaction of the excited Rydberg level $(|64D_{3/2}, -3/2\rangle + |64D_{5/2}, -3/2\rangle)/\sqrt{2}$ is plotted in (b) where z is perpendicular to the lattice plane.

$(|64D_{3/2}, -3/2\rangle + |64D_{5/2}, -3/2\rangle)/\sqrt{2}$. The two-color laser could be obtained in a setup of beamsplitters and acusto-optical modulators. The spatial profile of the Rydberg-Fermi interaction relative to the position of plaquette atoms is plotted in Fig. S1b. The generated Rydberg superposition state mainly contains the $Y_{2,-2}$ spherical harmonic term, which concentrates the electron wave-function close to the lattice plane and enhances the interaction strength. The plaquette atoms experience an effective Rydberg-Fermi interaction that is averaged over their spatial profile. The scattering energy of Rydberg electron over the qubit-dependent Wannier state of the lth plaquette atom in the geometry of Fig. 1a of the main text with single site confinements of $\text{FWHM}_{x,y}$=20nm and FWHM_z=35nm would be quantified by Eq. 2 of the main text as

$$\begin{aligned}&\bar{V}_{\text{RF}|1_l\rangle} = 1.1\text{MHz}, \quad \text{MD}_{V_{\text{RF}|1_l\rangle}} = 0.14\text{MHz} \\ &\bar{V}_{\text{RF}|0_l\rangle} = 0.27\text{MHz}, \quad \text{MD}_{V_{\text{RF}|0_l\rangle}} = 0.16\text{MHz}\end{aligned} \quad \text{(S3)}$$

where the in-plane qubit-dependent lattice-shift of $D =$

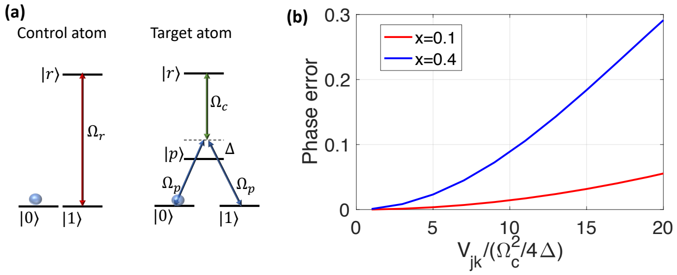

FIG. S2. Effects of unwanted intra-component interaction V_{jk} on the fidelity of conventional dipolar parallelized gate of [4]. (a) The level scheme for the cases with $|0_c\rangle$ states. Without the Rydberg control state, each target atom would acquire a partial Rydberg population of $(\Omega_p/\Omega_c)^2$. The unwanted interaction among target atoms generates an unwanted phase that suppresses fidelity. (b) The infidelity scales by the relative intra-component interaction strength as well as the Rydberg population $x = \sqrt{2}\max(\Omega_p)/\Omega_c$, see [4] for the applied pulse shape. The proposed Ryd-Fermi scheme in this article is an alternative with no cross-talk among target atoms $V_{jk} = 0$.

34.5nm is considered. The same Rydberg state in the geometry of Fig. 2c of the main text reduces the unwanted level shift of the $|0\rangle$ qubit state to

$$\bar{V}_{\mathrm{RF}|0_l\rangle} = 0.16\mathrm{MHz}, \quad \mathrm{MD}_{V_{\mathrm{RF}|0_l\rangle}} = 0.09\mathrm{MHz} \tag{S4}$$

with the qubit-dependent lattice shift of $D_z = 150$nm being perpendicular to the lattice plane.

SUPPLEMENTARY NOTE 2: LASER EXCITATION OF THE MOTIONAL STATES IN THE OPTICAL LATTICE

The laser excitation of atoms to the Rydberg state could lead to phases that depend on the atomic position. This could excite the motional states in the optical lattice. Let us consider the targeted atom in electronic and motional state $|1_e, 0_m\rangle$. The spatial variation of the two-photon excitation with the counter-propagating 1013nm and 420nm lasers is given by $e^{ik\hat{z}}$ with $k = k_{1013} - k_{420}$. We can rewrite the vibrations of the position operator as $\hat{z} = \sigma/2(\hat{a}_j^\dagger + \hat{a}_j)$, where $\sigma = \sqrt{\frac{\hbar}{m\omega_{tr}}}$ is the spread of the ground motional state wave-function, ω_{tr} is the trap frequency and $(\hat{a}, \hat{a}^\dagger)$ are the phononic annihilation-creation operators of the targeted site. In the Lamb-Dicke regime ($\eta = k\sigma/2 \ll 1$) one can expand the exponential to get

$$e^{ik\hat{z}} = (l + i\eta(\hat{a} + \hat{a}^\dagger) + O(\eta^2)). \tag{S5}$$

The Hamiltonian describing the laser excitation can now be written in the new basis $|1_e, 0_m\rangle$, $|r_e, 0_m\rangle$, $|r_e, 1_m\rangle$ as:

$$\begin{pmatrix} 0 & \Omega_r & \eta\Omega_r \\ \Omega_r & 0 & 0 \\ \eta\Omega_r & 0 & \omega_{tr} \end{pmatrix} \begin{pmatrix} |1_e, 0_m\rangle \\ |r_e, 0_m\rangle \\ |r_e, 1_m\rangle \end{pmatrix} \tag{S6}$$

Considering the setups described before Eq. 5 and Eq. 6 of the main text with FWHM$_z$=35nm, the probability of exciting a motional state $|r_e, 1_m\rangle$ over the Toffoli and fan-out operations with $\Omega_r/2\pi = 30$ kHz and 30MHz would be 0.3% and 1.5% respectively.

SUPPLEMENTARY NOTE 3: EFFECTS OF INTRA-COMPONANT INTERACTIONS IN C-NOT^k DIPOLAR GATE

The implementation of Rydberg-dipolar parallelized C-NOT^k gate [4] is sensitive to the intra-component interaction. The level scheme shown in Fig. S2a, considers the case of $|0_c\rangle$ state where each of the target atoms would follow the dark state $|D\rangle = (\Omega_c|0/1\rangle - \Omega_p|R\rangle)/N$ with the Rydberg population of $P_R = (\frac{\Omega_p}{\Omega_c})^2$ on each target atom. Applying the phase-dependent definition of fidelity in Eq. 10 of the main text, the dipolar Rydberg gate [4] shows significant sensitivity to intra-component interaction V_{jk} as shown in Fig. S2b.

SUPPLEMENTARY REFERENCES

[1] Weitenberg, Christof, et al. "Single-spin addressing in an atomic Mott insulator." *Nature* **471** 319 (2011).

[2] Mandel, Olaf, et al. "Controlled collisions for multi-particle entanglement of optically trapped atoms." Nature 425, 937 (2003).

[3] H. Levine, et al., High-fidelity control and entanglement of Rydberg-atom qubits, Phys. Rev. Lett. **121**, 123603 (2018).

[4] Müller, M., et al. Mesoscopic Rydberg gate based on electromagnetically induced transparency. *Phys. rev. let.* **102**, 170502 (2009).
